# Generated outcomes in risky choice reveal biased sampling and sequential dependencies

**DOI:** 10.1038/s44271-026-00467-y

**Published:** 2026-05-07

**Authors:** Jake Spicer, Yun-Xiao Li, Lucas Castillo, Johanna K. Falbén, C. Stella Qian, Adam N. Sanborn

**Affiliations:** 1 Department of Psychology, University of Warwick, Coventry, UK; 2 Department of Psychology, University of Amsterdam, Amsterdam, Netherlands; 3 Leibniz Institute for Psychology (ZPID), Trier, Germany; 4 Department of Computer Science, Texas State University, San Marcos, TX USA

**Keywords:** Human behaviour, Economics

## Abstract

Recent decision-making models have explained behaviour using *mental sampling* mechanisms, but there is still little agreement on the specific sampling process, such as whether sampling rates match true probabilities. Here, we seek to trace the sampling process using *generation* tasks: in two experiments using general online samples (*N*s = 52, 51), participants repeatedly produced potential outcomes from pairs of monetary gambles before choosing between them. Results found over-generation of rarer outcomes and under-generation of common outcomes overall, but not in initial responses, as well as avoidance of direct repetitions. Participants also tended to select options with higher average utility across their responses, implying generations guided choice. These findings suggest systematic biases in the information people may consider before a choice, and the influence that this can have on subsequent decisions, carrying implications for mental sampling models of this behaviour. We thus suggest explicit generation is a valuable method to access underlying choice processes, offering new assessments of existing theories of decision making.

## Abstract (web summary)

This study examined what information people may consider before a choice by asking them to repeatedly generate its possible outcomes. Participants overgenerated rare events and avoided direct repetitions, suggesting biases in what comes to mind.

## Introduction

How do we decide between options whose outcomes are uncertain or unknown? In modern decision-making research, the increasingly common answer is *mental sampling*, in which decision makers repeatedly generate potential outcomes of a choice in their mind and use these imagined events to direct selection^1–7^. These models commonly use sampling techniques to reduce complex choice environments into a small set of concrete examples to simplify decisions, and have shown substantial success in predicting choice behaviour, including several previously observed biases^3,8–10^.

Despite this common focus on sequential sampling, existing models differ substantially in their assumptions on exactly how such sampling operates. For example, Decision Field Theory (DFT) suggests sampling reflects stochastic allocation of attention towards possible outcomes, where allocation rates are proportional to the probability of that outcome occurring; that is, an event with an 80% chance of happening has an 80% chance of receiving attention^1^. Each sample is then integrated into an estimate of the value of that option, continuing until the difference in values between alternatives exceeds some threshold. A more recent variant of this model, DFT_*e*_, expanded this process by mixing the true probabilities with a uniform distribution across possible outcomes, reflecting instances where the decision maker is distracted and falls back on equal allocation of attention^11^.

A quite different sampling process is proposed by Decision by Sampling (DBS), in which a choice’s possible outcomes are compared against events sampled from memory to estimate their relative rank; for example, if an outcome is better than four of five retrieved instances, its rank is 0.8^2^. These ranks then offer a common currency which can be used to compare alternatives, but are shaped by the mental distribution of the decision maker’s collected experiences.

Utility Weighted Sampling (UWS) also suggests a sequential sampling process, but one based in resource rationality: within a limited number of samples, UWS proposes that particularly impactful outcomes (e.g., lottery wins, fatal accidents) should be over-represented to ensure they are not overlooked, meaning sampling rates are shaped by both an outcome’s probability and its value^4^.

A more complex sampling process is suggested by ‘Best Estimate and Sampling Tools’ (BEAST), which uses multiple sampling methods, stochastically selecting between them with each sample. These ‘sampling tools’ reflect differing behavioural motivations: samples may be unbiased draws from the true distribution, taken from a uniform distribution across possible events to reflect equal weighting, pessimistically focus on the worst possible outcome of each option, or focus on the signs of the possible outcomes rather than their specific values. These samples are then combined with the true expected value of that alternative to estimate each option’s value, with selections based on these estimates^3^.

Query Theory, meanwhile, offers a more qualitative sampling process in which decision makers pose themselves a series of questions regarding their choice and samples reflect answers that support particular selections. Crucially, the order of these queries is dependent on the context of the decision, and earlier queries can interfere with later ones, leading to differing behaviour according to which question is answered first: for example, buyers first consider the downsides of a purchase, leading to lower valuations, while sellers begin with the advantages, resulting in higher prices^5^.

Finally, Drift Diffusion Models (DDMs) offer a more abstract version of sampling, depicting choice as a diffusion process: decision makers begin ‘between’ the possible options, and each sample pulls them towards a particular choice, with the ‘drift’ capturing the general trend of movement. As samples accumulate, the decision maker eventually crosses a decision threshold, with the number of samples then dictating the response time^6,7^.

As this should illustrate, while these models all assume choices are made through mental sampling, there is little agreement on the exact algorithm used. This is particularly apparent in their assumptions on the rates at which outcomes are sampled: models differ on whether sampling rates match objective probabilities (DFT) or are biased towards uniformity (DFT_*e*_, BEAST), whether sampling is influenced by an outcome’s value (UWS), and whether sampling involves contrasts with previously observed cases (DBS). Models also disagree on whether sampling is independent (DFT, BEAST) or autocorrelated (Query Theory), and whether decision makers take a fixed number of samples before making a choice (UWS, BEAST) or continue sampling until the collected evidence definitively favours one option (DFT, DDMs).

These differences are not purely theoretical but have meaningful implications on behaviour: biases in sampling rates can skew subsequent choices by over- or under-representing certain outcomes, leading to potential risk or loss aversion^4,11^. Autocorrelated sampling can lead to more homogeneous evidence where new samples remain close to their predecessors, as well as potential biases towards the initial sample under low sample counts^12,13^. Evidence-based stopping rules meanwhile, create connections between choices and the time taken to make a decision, for example, predicting longer response times for more difficult choices where evidence is more conflicted^7,14^. There is thus a need for greater detail on the sampling process used in decision-making to separate these theories.

Previous attempts to distinguish these mechanisms have predominantly focused on choices themselves, inferring the underlying decision process from particular preference patterns or fitting models to selections^3,9–11,15,16^. While such methods certainly point towards the systems likely to support choice, these are a step removed from any actual sampling, observing only the final result. This also creates difficulty in separating the influence of differing aspects of the alternatives on choice, for example, whether outcomes are sampled purely according to their probability or also their value. An alternative approach is to attempt to trace the underlying sampling process using targeted experimental methods. For example, studies have asked participants to explicitly state their relevant thoughts during choice^5,17^, while other work has used eye-tracking to measure attention towards each option as a proxy for its consideration^8,18,19^. These methods also provide key insight into the decision-making process, but are more limited in what they can say about existing models: thought-listing gives an indication of what comes to mind but is more qualitative than the numerical values commonly assumed in theories of decision making, while eye-tracking might reflect attention towards an option but not how that option is evaluated.

One promising method to trace the mental sampling process is *random generation*, in which participants are asked to repeatedly produce items from a given set in a random manner, for example, randomly producing numbers between 1 and 10. Previous studies have used these tasks to test people’s ability to emulate true randomness, where items are drawn independently and with equal probability^20–22^. This work has often found systematic deviations from these properties in human-generated sequences: in particular, people commonly fail to produce independent series, demonstrating predictable patterns in their responses^21–25^. Similar tasks have also been used in competitive settings where players can select between multiple actions: in these cases, random selection helps avoid predictable strategies which can be exploited by opponents^26,27^. A simple example is Rock–Paper–Scissors: players who always pick the same option are easily beaten, whereas random selection cannot be anticipated, in fact, providing the optimal strategy. These selection probabilities need not be equal to be optimal, however, as adaptive patterns can vary according to the payoff structure: more valuable actions may be chosen more often, so long as this does not create an advantage for the opponent^26^.

The advantage of random generation in choice settings is that this procedure resembles the mental sampling assumed in the decision-making theories noted above: the set of potential outcomes of a choice form a probability distribution, and decision makers iteratively produce a series of instances from this distribution to guide their selection. Applying the random generation paradigm to choice may then offer a method to externalise the internal sampling process for more direct examination of the underlying system: while actual mental samples are ultimately unobservable, asking decision makers to explicitly sample events could provide insight into the accessibility of outcomes in the mind, and the properties of the algorithm used to draw them. In this case, the focus is not specifically on people’s randomness but the broader statistical signatures of the generating process, including deviations from the given distribution (whether uniform or not) and sequential dependencies between responses, as well as relations to subsequent judgments. This falls in line with a series of recent studies using similar generation tasks to access people’s representations of distributions in a broad range of domains, from simple digits^28^ to environmental distributions like people’s heights or lifespans^29,30^ to stereotypes about social categories^31^. This includes previous applications to choice settings, which asked participants to generate new examples of available choice options after limited observations to infer their assumed distribution^32^. Results from these tasks have provided evidence of serial dependencies in people’s generation procedure^29,30,33^, systematic deviations from objective probabilities with particular biases in initial responses^28,31^, as well as links between generated samples and related judgments^30–32^.

Applying generation tasks to decision making thus offers a fruitful approach for evaluating existing decision theories by eliciting the samples they commonly assume and testing for predicted patterns such as over-representation of rare or extreme outcomes. This is not to suggest that explicit generations are exact reports of mental samples, but rather that generated sequences might be *representative* of the true sampling process, revealing key biases in the accessibility of evidence that could influence choice. Such tasks also allow for assessments of this link by comparing generations with subsequent decisions: if explicit generations capture similar sampling processes to those guiding choices, then these responses could be used to predict selections. These comparisons then offer a test of the method itself, assessing whether explicit generations provide additional insight into the choice process.

The current paper thus uses explicit generation tasks to attempt to access the sampling process underlying decision-making to evaluate the assumptions of existing mental sampling models. We report two experiments applying generation tasks to the common decision-making paradigm of risky choice: participants were presented with pairs of monetary gambles and repeatedly produced their possible outcomes before choosing between them. These generated sequences were then examined for key patterns suggested by proposed decision-making models, as well as their relation to subsequent choices. Based on mechanisms proposed in previous mental sampling models, we examined three particular research questions regarding people’s generated values:

- **RQ1** Do the rates at which participants generate outcomes follow their true probabilities (DFT), or are these systematically biased (DFT_*e*_, BEAST)?
- **RQ2** Is the generation rate of an outcome influenced by its value (UWS)?
- **RQ3** Are generated outcomes independent of one another (DFT, BEAST, UWS), or are there sequential dependencies within the series (Query Theory)?

In addition, we examined a fourth question reflecting a common assumption across these models:

- **RQ4** Do choices correspond with the evidence collected across generations?

## Methods

In two experiments, participants viewed a series of pairs of monetary gambles comparing a more variable, riskier prospect against a safer or certain alternative. For each pair, participants repeatedly generated potential outcomes from both gambles, before choosing the gamble they would prefer to play. Experiment 2 differed from Experiment 1 only in that participants also chose between options before generating any outcomes as well as after to test for any change in preference following generation. All experiments received ethical approval following review by the University of Warwick Humanities and Social Sciences Research Ethics Committee (Ref: HSSREC 188/23-24). Anonymised data and analysis code are available on the Open Science Framework (https://osf.io/f5wje/). Neither Experiment 1 nor Experiment 2 was preregistered.

### Experiment 1

#### Participants

Fifty-two participants (28 female, 24 male; age range 20-79 years, *M* = 38; 81% White, 8% Black, 8% Asian, 4% Other) were recruited from Prolific Academic in return for £4 in payment plus a variable bonus (£0–£12.80) depending on choices in the task, detailed below. We aimed for a sample size of 50 participants to roughly correspond with counts used in previous generation studies^29,30^. Post-hoc power analyses using G*Power 3.1^34^ found that this count provides a minimum power of 0.934 to detect medium effect sizes across our performed analyses. Participants were required to live in the UK to ensure familiarity with the currency used in the gambles, and had completed at least 100 previous submissions on the Prolific platform with an approval rating of at least 95%. Demographic information was provided separately by Prolific and not attached to individual responses. No information was collected on socioeconomic status or communities of descent. All participants consented to take part in the study following an explanation of the task and the intended use of their data.

#### Design and materials

Experiment 1 used the risky choice paradigm, used extensively in past decision making research^15,19,35–39^, including previous assessments of mental sampling models^1,3,9–11^. Participants were presented with pairs of monetary gambles with set probabilities of winning or losing particular amounts of money. We chose to present gambles through written description (as opposed to via experience with actual outcomes) to reduce the influence of memory on generations, focusing more directly on the underlying sampling algorithm from a specified distribution. 8 gamble pairs were selected from Erev et al.^3^, intended to test key choice phenomena including risk and loss aversion, overweighting of rare events and splitting of gains. Gamble descriptions are given in Table 1, with more detailed outlines of each effect given in Supplementary Note 1. Note that in these pairs, option B offers the riskier prospect, while option A provides certain or safer outcomes.

**Table 1:** Gamble pairs used in Experiments 1 and 2, taken from Erev et al.^3^, and the effects they test (note that ‘Certainty’ and ‘Splitting’ contrast choice between 2 pairs rather than within a pair)

ID	Effect	A	B	Expected preference	Observed B choice rate
1	Certainty	£3, 1	£4, 0.8 £0, 0.2	A > B in Pair 1, B > A in Pair 2	44.0%
2		£3, 0.25 £0, 0.75	£4, 0.2 £0, 0.8		58.4%
3	Reflection	−£3, 1	−£4, 0.8 £0, 0.2	B > A	50.4%
4	Overweighting of rare events	£2, 1	£1, 0.99 £101, 0.01	B > A	52.0%
5	Loss aversion	£0, 1	£50, 0.5 -£50, 0.5	A > B	38.4%
6	Risk aversion	£9, 1	£2, 0.5 £4, 0.25 £8, 0.125 £16, 0.0625 £32, 0.0313 £64, 0.0156 £128, 0.0078 £256, 0.0078	A > B	37.6%
7	Splitting	£16, 1	£1, 0.6 £50, 0.4	B in Pair 8 >B in Pair 7	49.6%
8		£16, 1	£1, 0.6 £44, 0.1 £48, 0.1 £50, 0.2		52.0%

Gambles are formatted as [*o*, *p*], where *p* is the probability of receiving outcome *o*, and each line reflects a unique possible outcome. Expected preference gives the choice pattern corresponding to that effect, while Observed B Choice Rate gives the percentage of participants choosing B in that gamble in Erev et al.^3^.

We recorded two main types of data during the experiment: first, the series of outcomes generated by a participant for each gamble, and second, their preferred option within each gamble pair. We then used these data to produce multiple dependent variables to investigate our research questions, including the rate at which each outcome was generated within a sequence, measures of sequential dependencies between responses, and relations between generated series and subsequent choices, detailed further in the Results section below. Post-hoc tests found these measures, in some cases, deviated from assumptions of normality made by our analyses, most notably the generation rates, which displayed heavy tails in regression residuals. This is likely due to these rates being restricted between 0 and 1; while this could be addressed by rescaling the data, this commonly additionally requires filtering values at or below zero, which risks biasing the data and so is avoided here. This should not affect calculated means or coefficients, but may lead to imprecision in inferential statistics, suggesting some caution in their interpretation. The major independent variables of the task were the gamble parameters given by the descriptions: the values of each possible outcome and their true probability.

#### Procedure

Experiment 1 asked participants to generate outcomes from a series of monetary gambles as if they were repeatedly played out. To aid intuition, participants were told they could view each gamble as a jar of balls, each having a monetary value written on it, with the gamble description listing the proportions of each ball in the jar; generations were then framed as imagined draws from the jar, including replacement and reshuffling between each response.

**Fig. 1:**
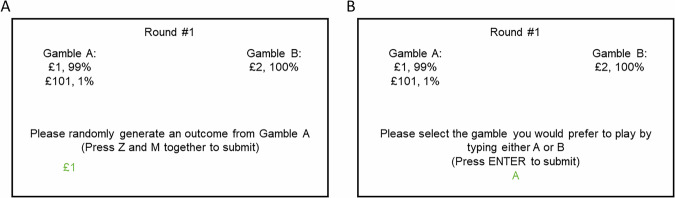
Experimental design. Illustrations of the experimental procedure, showing a generation trial (**A**) and a choice trial (**B**). Green text reflects participant responses.

The experiment was made up of 8 generation trials reflecting the 8 gamble pairs presented in a randomised order, as well as one practice trial at the start of the experiment to introduce participants to the generation task. Figure 1 illustrates the experimental procedure. In each trial, participants were presented with the two alternatives for that choice on either side of the screen. In case of biases towards particular positions, assignment of alternatives to the sides was randomised, though the left alternative was always labelled as ‘Gamble A’ and the right alternative as ‘Gamble B’, independent of their label in Table 1.

Participants gave their responses by typing each imagined outcome using the number keys and submitting their response by simultaneously pressing the ‘Z’ and ‘M’ keys; this key combination was used to force participants to move their fingers away from the number keys between responses to discourage them from simply repeatedly hitting the same key. We used typed responses to ensure participants were aware of the outcomes’ values during generations. While participants were free to type any value, the experiment only allowed submission of the listed outcomes for that gamble. To ensure an equal number of responses were recorded for each gamble within a pair, participants were made to alternate generations between the two options (i.e., A, B, A, B...). Generations continued for 2 min per pair. Participants could respond at their own pace, but a time limit of 60 s was placed on each response to ensure task progression. We used a fixed time limit for generations to balance the number of responses collected against the length of the task: allowing participants to terminate generation themselves might severely limit the number of responses recorded, while pre-set sample counts might prolong the task and introduce fatigue.

After generations for a given gamble pair were complete, participants were asked to select which of the two gambles they would prefer to play by typing either ‘A’ or ‘B’ and pressing ‘Enter’ to confirm their choice. To incentivise reporting of true preferences, participants were told at the start of the experiment that one of their selected gambles would be randomly selected at the end of the experiment and played for real to determine their bonus payment.

Once all trials were complete, one of the participants’ chosen gambles was randomly selected, and an outcome was sampled according to the stated probabilities. Participants then received 5% of that outcome as a bonus payment; participants were informed of this scaling at the start of the task, though not the specific rate. If the selected outcome was negative, the bonus was zero. Finally, participants were debriefed on the aims and expectations of the study. The task took an average of 25 min to complete, and the average bonus was £0.33 (range: £0–£2.50).

### Experiment 2

#### Participants

Fifty-one participants (28 female, 22 male, 1 non-disclosed; age range 21–68 years, *M* = 40; 78% White, 8% Mixed, 2% Black, 2% Asian, 4% Other, 6% non-disclosed) were recruited from Prolific Academic in return for £4 in payment plus a variable bonus (£0–£12.80) depending on choices in the task. Sample size was set to roughly match that of Experiment 1. Selection criteria were identical to Experiment 1, with the addition that participants must not have taken part in the previous experiment. Demographics were again provided by Prolific separately from the experimental data. All participants consented to take part in the experiment after an explanation of the task and the intended use of their data.

#### Design, materials and procedure

Experiment 2 was identical to Experiment 1 except for one aspect: before performing any generation trials, participants first selected between all 8 gamble pairs in an initial choice block. This provided a within-subjects comparison of choice preference before and after generations to examine any influence of producing outcomes. Choices were made in the same manner as the post-generation choices described in Experiment 1 above. The task took an average of 27 min to complete, and the average bonus was £0.41 (range: £0–£2.50).

## Results

The same set of analyses was applied to data from both experiments, with the addition of comparisons of pre- and post-generation choice for Experiment 2; we therefore report analyses for both tasks together here for concision. Analyses fall into three broad categories: first, tests of the rates at which each gamble outcome was generated compared to its true probability and value; second, examinations of sequential dependencies within a participant’s series of generations for a gamble; and third, the relation between generations and the subsequent choice between the two gambles in that pair. Note that within the following analyses, all reported tests are two-sided.

### Exclusions

Data was first screened to ensure no invalid outcomes were accidentally included in the responses: this only occurred if a participant timed out for a generation after typing but not submitting a response. Any generated values not possible in that gamble were replaced with a non-number filler to preserve response order, eliminating 12 (0.05%) responses in Experiment 1 and 38 (0.19%) in Experiment 2.

We also removed participants who appeared to be solely generating the best outcomes from each gamble, potentially under the mistaken belief that this would increase their bonus. The criterion for this was set at the generation of only the highest outcome for the riskier gamble in more than half of the trials, which led to the exclusion of 1 participant in Experiment 1 and 5 participants in Experiment 2.

### Generation rates

The mean generation count per gamble was 26.7 in Experiment 1 and 24.0 in Experiment 2 (53.4 and 48.0 responses per pair, respectively), though there was substantial variation in this count between participants in both tasks (E1: standard deviation = 13.0; E2: SD = 13.4) due to the self-pacing of generations. Analyses of participant generations were restricted to only responses for the riskier gambles (i.e., those labelled B in Table 1) as generations for gambles with singular outcomes are necessarily constant (as enforced by our task and exclusion criteria).

**Fig. 2:**
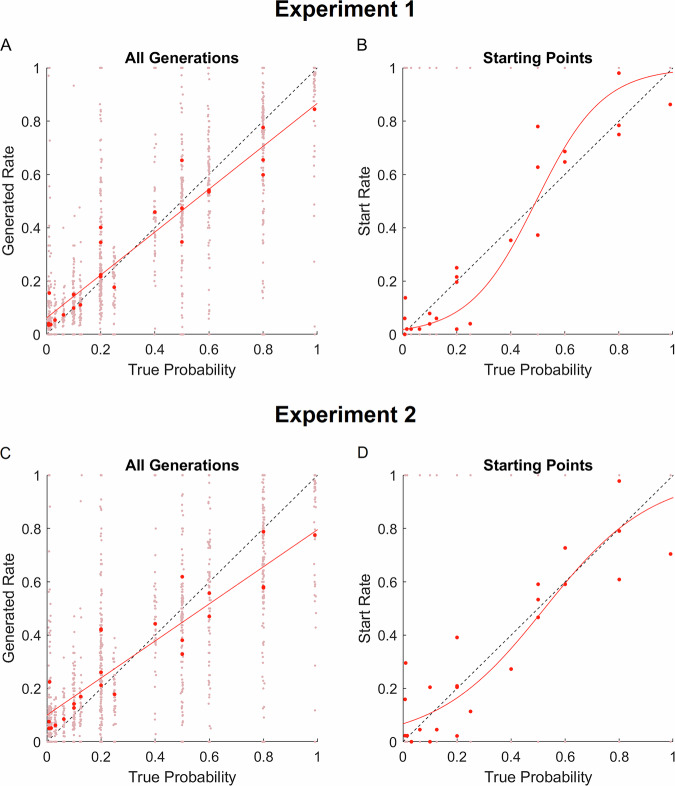
Generation rates. Generation results from Experiments 1 and 2 showing both overall generation rates (left column) and rates for the first response for each gamble (‘Start Rate’; right column). Light points represent individual-level rates and bold points represent means across participants (*n* = 51 participants in Experiment 1 and *n* = 46 participants in Experiment 2). Red lines represent regression fits across individual data (*n* = 1218 rates in Experiment 1; *n* = 1088 rates in Experiment 2), while dotted lines show equality.

We began by examining the rate at which each gamble outcome was generated, given its described probability and value, targeting RQ1 and RQ2. For each possible outcome in each gamble, we calculated the relative frequency of that outcome in each participant’s generated series to measure their generation rate. These rates were passed to mixed-model linear regressions for each experiment using the predictors of outcome value and probability from the gamble descriptions, with random intercepts and slopes for both value and probability for each participant. Results are summarised in Table 2. These found a significant positive relationship between generated rates and stated probabilities in both tasks (E1: *β* = 0.80 [95% confidence interval: 0.72, 0.89], *t*(1215) = 18.0, *p* < . 001; E2: *β* = 0.70 [0.60, 0.79], *t*(1085) = 14.1, *p* < . 001), suggesting generations did broadly follow gamble descriptions, as illustrated in Fig. 2A, C. To determine whether participants accurately followed objective probabilities, we subsequently compared these slopes against 1, reflecting perfect alignment; this found slopes in both tasks were significantly below this standard (E1: *t*(1215) = −4.37, *p* < . 001; E2: *t*(1085) = −6.18, *p* < . 001), implying a general bias in generation rates: low probability events were over-generated and high probability events were under-generated.

**Table 2:** Generation rate mixed-model regression results across all generations and starting points only in each experiment

Dataset	Experiment	Predictor	*β*	*t*	DF	*p*
All generations	1	Intercept	0.06 (0.03, 0.10)	3.58	1215	<0.001
		Probability	0.80 (0.72, 0.89)	18.0	1215	<0.001
		Value	8.73e-05 (−0.0001, 0.0003)	0.81	1215	0.416
	2	Intercept	0.10 (0.06, 0.14)	5.43	1085	<0.001
		Probability	0.70 (0.60, 0.79)	14.1	1085	<0.001
		Value	7.45e-05 (−0.0002, 0.0003)	0.61	1085	0.539
Starting Points	1	Intercept	−3.99 (−4.73, −3.26)	10.6	1207	<0.001
		Probability	8.09 (6.46, 9.72)	9.76	1207	<0.001
		Value	0.004 (−0.001, 0.008)	1.67	1207	0.096
	2	Intercept	−2.63 (−3.05, −2.21)	12.2	1061	<0.001
		Probability	5.02 (4.08, 5.96)	10.5	1061	<0.001
		Value	−0.0006 (−0.005, 0.004)	0.28	1061	0.778

*β* gives coefficient estimates for that predictor with brackets giving 95% confidence intervals, and *t*, DF and *p* measure whether this coefficient significantly differs from zero.

In contrast, both models found no significant effect of an outcome’s value on its generation rate (E1: ∣*β*∣ < 0.0001 [−0.0001, 0.0003], *t*(1215) = 0.81, *p* = 0.416; E2: ∣*β*∣ < 0.0001 [−0.0002, 0.0003], *t*(1085) = 0.61, *p* = 0.539), providing no evidence of a value-based bias in either experiment. Moreover, the estimated effect size of value is minuscule: outcomes would need to exceed £10,000 to cause a difference of 1 percentage point in generation rate. Interestingly, this can still mean certain values are over-represented due to negative correlations between outcome value and probability in the gamble set: larger outcomes tend to have lower probabilities, meaning over-generation of rarer events also favours these extreme values. A supplementary analysis (discussed further in Supplementary Note 2) found this was indeed shown in these data: generation rates for higher outcomes tended to exceed their true probability, while rates for lower outcomes fell below their true probability. Even so, the regression results provided above find no significant influence of outcome value on generation rate once such correlations between value and probability are considered, suggesting that these effects are ultimately driven by biases in probability rather than value itself.

#### Starting point

We also specifically examined which outcome participants generated first for each alternative, drawing on recent suggestions that early responses in generation tasks can show particular biases towards more likely or typical events^28,31,40^. For each outcome in each gamble, we created a new binary variable indicating whether that outcome was the first generated for each participant, with mean rates across participants summarised in Fig. 2B, D. These binary values were then passed to mixed-model logistic regressions for each experiment using outcome value and true probability as predictors, again including random intercepts and slopes for both predictors for each participant. Results from these regressions are also summarised in Table 2: participants were more likely to begin with higher probability outcomes in both tasks (E1: *β* = 8.09 [6.46, 9.72], *t*(1207) = 9.76, *p* < 0.001; E2: *β* = 5.02 [4.08, 5.96], *t*(1061) = 10.5, *p* < 0.001), again suggesting sensitivity to objective probabilities.

To compare this relationship against perfect alignment with objective probabilities, we ran separate linear regressions for each experiment on the mean start rate across participants for each outcome from each gamble, though this substantially reduced the data count. This found no significant difference from a slope of 1 in either task (E1: *β* = 1.10 [0.93, 1.27], *t*(21) = 1.18, *p* = 0.251; E2: *β* = 0.93 [0.72, 1.13], *t*(21) = −0.77, *p* = 0.451), conflicting with the above finding that slopes across all generations fell below 1. In addition, the confidence intervals for these starting point slopes do not overlap with the slope estimates found above for overall generations, suggesting distinct effects of probability in the first response.

As with the overall generation rate, however, the start rate showed no significant influence of outcome value in either experiment (E1: *β* = 0.004 [−0.001, 0.008], *t*(1207) = 1.67, *p* = 0.096; E2: *β* = −0.0006 [−0.005, 0.004], *t*(1061) = −0.28, *p* = 0.778), meaning starting points show no evidence of any reliable bias.

### Sequential dependencies

We next examined the generated sequences for sequential dependencies to answer RQ3, again restricting attention to the riskier gambles. Dependencies were assessed using three measures: the *autocorrelation function (ACF)* within each generated series, the rate of *repetition* across outcomes, and the *size of movements* between consecutive responses.

#### ACF

We first calculated the ACF for each sequence of responses for each gamble as a general measure of dependencies across generations. The ACF measures the correlation coefficient between a series and its lagged variant:

1 $${\mathrm{ACF}}(t)={\mathrm{Corr}}({x}_{i},{x}_{i+t})$$

where *x* is the series and *t* is the considered lag. Independent series are thus expected to show ACF coefficients of 0, with deviations from this standard suggesting sequential dependencies. Given the relatively short length of the generated sequences, we focus on lags of 1 to 10 to ensure sufficient data for reliable estimates.

**Fig. 3:**
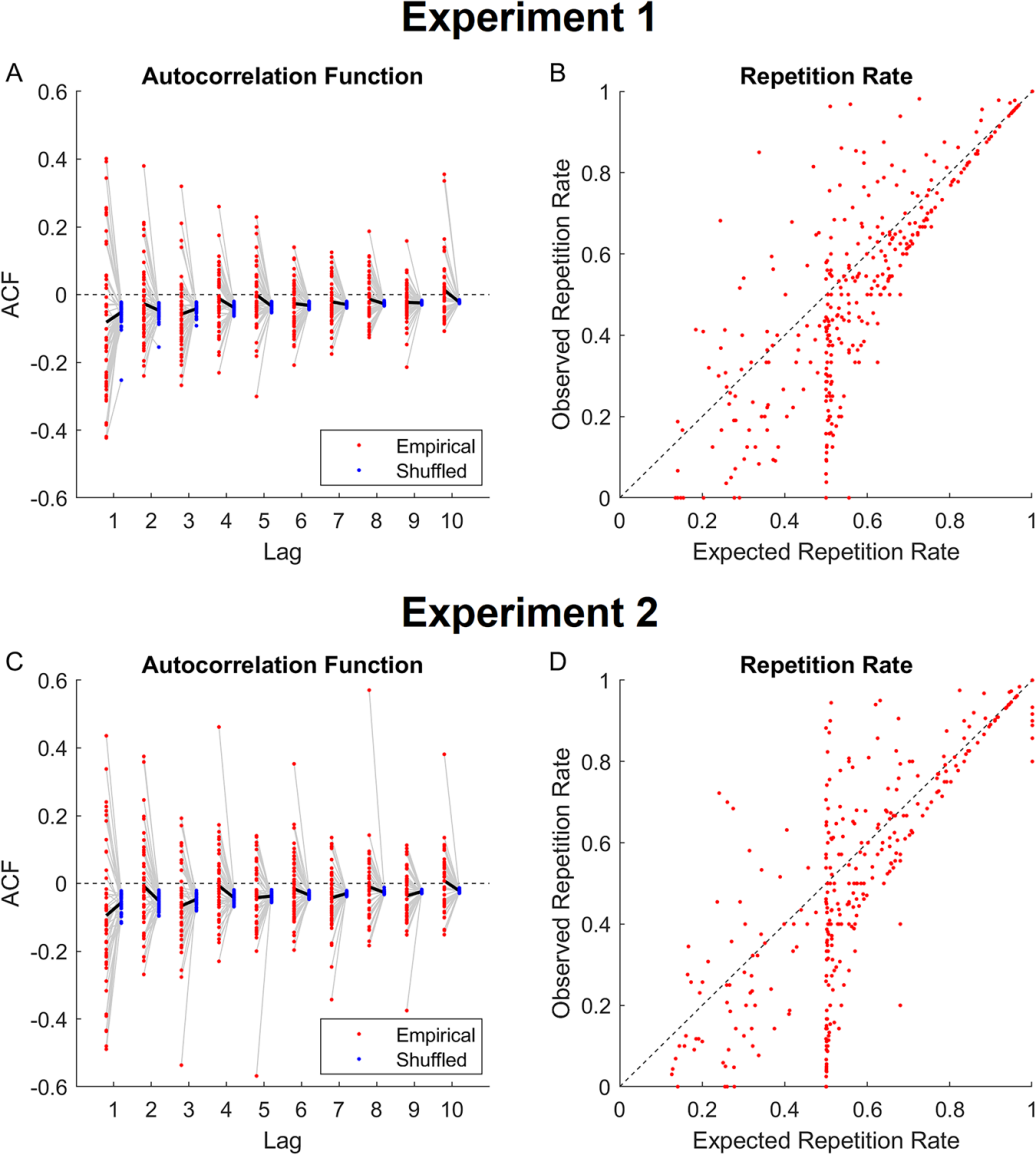
Sequential dependency results. Sequential dependency measures from Experiments 1 and 2. Panels **A** and **C** show the mean ACF for each participant across sequences from each task (red points), as well as equivalent values from shuffled series reflecting expectations under independence (blue points). *n* = 8 coefficients per point in both experiments. Grey lines connect common participants, while black lines give averages. Panels **B** and **D** show observed repetition rates against expected repetition rates assuming independence.

Mean ACFs for Experiments 1 and 2 are illustrated in Fig. 3A, C, respectively, showing many participants deviated from the theoretical independent standard of zero in both tasks, particularly at shorter lags. Deviations from this standard may not necessarily indicate true deviations from independence, however, as ACF coefficients could be biased due to the relatively short length of these series. We therefore calculated a more specific baseline for independence for each generated series by taking equivalent ACF coefficients from 1000 shuffled versions, ensuring comparisons have matching sequence length and outcome proportions but eliminating sequential dependencies. Mean ACF coefficients were then taken across shuffles to provide independent expectations for that series. We then calculated the difference between each empirical ACF coefficient and its expected value under independence for that participant and gamble and passed these differences to mixed-model regressions for each experiment with each lag as a predictor and random intercepts and slopes for each participant. In case of nonlinearities in deviation between lags (see Fig. 3), each lag was treated as a distinct categorical predictor rather than a single continuous variable. This found no significant deviations from independence in the observed series in either experiment: while the mean empirical ACF is slightly negative at the shorter lags in both tasks, similar negative biases are also shown in the shuffled series due to their short length. Full results from this analysis are given in Tables S1 and S2 in Supplementary Note 3.

We also used the ACFs to perform Ljung-Box tests^41^, which provide an aggregate statistic *Q* reflecting autocorrelations across lags for inferential testing:

2 $$Q=n(n+2)\mathop{\sum }\limits_{k=1}^{h}\frac{{\rho }_{k}^{2}}{n-k}$$

where *n* is the length of the sequence, *ρ*_*k*_ is the ACF at lag *k*, and *h* is the maximum evaluated lag. By this measure, 28.9% of generated sequences in Experiment 1 and 25.2% in Experiment 2 displayed significant evidence of autocorrelation, with Pair 5 having the highest rate in both cases (E1: 40.9%; E2: 41.3%), while Pair 6 held the lowest in Experiment 1 (13.0%) and Pairs 1 and 4 had the joint lowest in Experiment 2 (15.2%). This may then indicate some variation in autocorrelations between participants and gambles, which is masked in the aggregated ACF results.

#### Repetitions

We next examined the rate of repetitions in successive responses based on findings of apparent aversion to repetitions in sequential responses in past studies of random generation^23,29,42^. To investigate this, we compared the observed repetition rate from each generated sequence against that expected under independence. Expected repetition rates were defined by the sum of the squared generation rates, assuming the probability of repeating each outcome is the same as originally generating it. Repetition rates are illustrated in Fig. 3B, D. Paired *t*-tests found participants indeed under-repeated (E1: *M* = 57.0%; E2: 54.0%) compared to independent expectations (E1: *M* = 62.9%; E2: 60.0%) in both tasks, with an average difference of −5.85% [−7.05%, −4.19%] in Experiment 1 (*t*(50) = 4.63, *p* < .001, Cohen’s *d* = 0.63 [0.46, 0.81]) and −6.02% [−7.75%, −4.29%] in Experiment 2 (*t*(45) = 4.37, *p* < .001, *d* = 0.64 [0.44, 0.85]).

#### Movement size

Finally, we examined the size of movements between successive responses to determine whether transitions between generated outcomes were influenced by the difference in their values: if shifts to closer values are preferred, then observed movements will be smaller than expected under independence. We restrict this analysis to only the gambles offering more than two outcomes (Pairs 6 and 8), as movements between responses are naturally restricted for binary cases. This is especially relevant given the low rate of repetitions noted above: when movements can only be repetitions or alternations, a low rate of repetitions necessitates that observed movements will be high.

For each generated sequence, we calculated the mean absolute difference between successive responses after filtering repetitions. We then produced equivalent expected distances under independence by taking the same measure from 1000 shuffles of each empirical series and averaging across these shuffled sequences. Differences between the observed and expected movements were then passed to intercept-only mixed-model regressions, including random intercepts for each participant. These found observed movements were significantly lower than shuffled sequences in both experiments (E1: *β* = −4.71 [−6.55, −2.93], *t*(92) = 5.21, *p* < .001; E2: *β* = −5.93 [−9.84, −2.02], *t*(86) = 3.02, *p* = .003), suggesting that while participants seemingly avoided repeating the same outcome, when a wider range of values was available, they preferred moving to more similar numbers.

### Choice rates

We next examined participants’ choices between options in each pair; for Experiment 2, this is separated into pre- and post-generation choice, examining behaviour both within each block, as well as comparing choices between them.

#### Replication of choice phenomena

We first examined whether choice rates in this experiment replicated effects from previous studies using similar problems, focusing specifically on the choice rates from Erev et al.^3^ from which the gambles were drawn. Comparisons of these rates showed replication of many of these effects, as summarised in Table 3: in both experiments, participants demonstrated aversion to uncertainty in gains (preferring gamble A in Pair 1) but not losses (preferring gamble B in Pair 3), overweighting of rare events (preferring gamble B in Pair 4), and aversion to risk (preferring gamble A in Pair 6). In contrast, participants did not replicate loss aversion (showing no aversion to gamble B in Pair 5) in either experiment, while splitting effects are conflicted: splitting is observed pre-generation in Experiment 2 (increased preference for gamble B in Pair 8 compared to Pair 7), but is not seen post-generation in this task or in Experiment 1. These differences could suggest the generation trials slightly altered choice behaviour, though this might also be due to differences in our participant sample (a general online sample rather than a university class) or in the currency used (British Pounds rather than Israeli Shekels). The absence of loss aversion in particular may be due to the lack of any realised losses in our reward scheme, as sampled losses were converted to a bonus of £0. Described losses may then not have had sufficient impact to demonstrate these effects, though notably this did not appear to prevent the reflection effect in Pair 3.

**Table 3:** Choice rates from Experiments 1 and 2, including comparison with equivalent rates from Erev et al.^3^

		B choice rate			
ID	Effect	Erev et al.^3^	Experiment 1	Experiment 2	
				Pre-generation	Post-generation
1	Certainty	44.0%	31.4%	23.9%	34.8%
2		58.4%	47.1%	50.0%	52.2%
3	Reflection	50.4%	58.8%	60.9%	71.7%
4	Overweighting of rare events	52.0%	64.7%	52.2%	69.6%
5	Loss aversion	38.4%	56.9%	58.7%	60.9%
6	Risk aversion	37.6%	27.5%	32.6%	39.1%
7	Splitting	49.6%	45.1%	23.9%	41.3%
8		52.0%	29.4%	47.8%	41.3%

Note that within this gamble set, B is the riskier option, meaning higher percentages reflect more risk seeking.

#### Pre- vs post-generation choice

We next specifically compared choices before and after generations in Experiment 2, finding a notable increase in risk-seeking for almost all gambles: preference for the riskier prospect was on average higher post-generation for all pairs except Pair 8. A paired *t*-test confirmed this increase in riskiness was significant, *t*(45) = 2.65, *p* = 0.011, *d* = 0.39 [0.15, 0.64]. This may be related to the apparent biases in participant generations detailed above: the over-representation of low probability events in generations will tend to favour the riskier prospects offering rare but large gains, thereby increasing the attractiveness of these options in the second choice block. This is especially relevant given that these biases are not seen in initial responses but only appear after extended generation: asking participants to consider more samples during generation may then have further highlighted the rarer events, amplifying this effect.

Some support for this suggestion can be found by running a similar comparison on the dataset of Erev et al.^3^, which also repeated choices for these specific gamble pairs but did not request generations in between: in this case, no significant difference in riskiness is observed (*t*(124) = 0.00, *p* = 1, *d* = 0.00 [−0.10, 0.10]), potentially indicating that choices can be altered by intervening generations, though this again may also be influenced by differences in participant sample and methodology.

#### Predicting choice from generations

We next investigated whether choices were influenced by generations by predicting participants’ preferences in each pair using their generated sequences, targeting RQ4. For Experiment 2, we focus here on post-generation choices as pre-generation choices are unlikely to have been influenced by these later responses, though we examine these choices further in Supplementary Note 4. To provide predicted choices, we applied a number of potential choice rules to the generated sequences, drawing on basic functions such as which alternative held the higher average generated outcome, as well as a number of heuristic rules by which selections may be made, proposed in the literature^36,38,39^. These heuristics have previously been applied to the stated values and probabilities from the description of each gamble, though here we instead take the generated rates in place of objective probabilities to assess the relationship between generation and choice. We focus on these simple rules as our generation task was not intended for fitting more complex decision models, providing only a small number of choices per participant. This also avoids comparisons between flexible models with adjustable parameters and simple inflexible heuristics, placing all candidates on relatively equal footing.

We considered a total of 12 potential rules, listed in Table 4, with all rules defined in Supplementary Note 4. While these rules can be used to predict discrete choices, we here accessed the underlying continuous measures used by these rules to compare options (for example, the average outcome generated for each gamble), taking the difference in these measures between options to allow for sensitivity to magnitude. These differences were then passed to separate mixed-model logistic regressions for each rule predicting choice of the riskier gamble based on its advantage in the considered measure (following from the previous example, the mean generated outcome for gamble B minus the mean generated outcome for gamble A). Regressions also included intercept terms to capture general risk aversion or seeking independent of gamble parameters, as well as random intercepts and slopes for each participant to reflect individual differences.

**Table 4:** Choice rule comparison results

	Experiment 1		Experiment 2	
Rule	Log likelihood	*LR*	Log likelihood	*LR*
Higher mean	−240.94	<0.001	−204.79	0.010
Expected utility	−226.47	1	−200.20	1
Probable	−236.47	<0.001	−207.66	0.001
Least likely	−255.23	<0.001	−206.86	0.001
Most likely	−238.07	<0.001	−206.95	0.001
Lexicographic	−238.23	<0.001	−203.28	0.046
Tallying	−245.14	<0.001	−201.73	0.216
Equiprobable	−250.08	<0.001	−204.52	0.013
Equal weight	−252.99	<0.001	−201.37	0.311
Better-than-average	−251.01	<0.001	−203.95	0.023
Minimax	−254.37	<0.001	−208.10	<0.001
Maximax	−254.61	<0.001	−205.97	0.003

Log likelihoods give fit to empirical data (higher values reflect better fits), while *LR*s give likelihood ratios between each rule and the best-fitting rule in that experiment (in both cases, expected utility).

To simplify this contrast, we restrict attention to the rules which were found to best map generations to decisions, as measured by their likelihood of producing the empirical choice data from their respective regression model. Within each experiment, we found the best-fitting rule by maximum likelihood, and then compared all other rules to this standard via likelihood ratios, filtering any rules with ratios below 1/3, the standard criterion for a substantial effect. Likelihoods and their ratios for all rules in each experiment are listed in Table 4, with further regression results given in Tables S3 and S4 in Supplementary Note 4.

This filtering led to a single best rule in each task: *expected utility*. This rule assesses the mean generated outcome following a conversion of objective values to subjective utilities, as has been commonly assumed in previous decision making research^1,11,35^. To reflect this conversion, we applied a basic concave utility function to generated values using a power law with exponent 0.5, based on similar findings in past studies^39^:

3 $${{\rm{E}}}[{{\rm{U}}}(x)]=\mathop{\sum }\limits_{i}p({x}_{i}){\mathrm{sign}}({x}_{i})| {x}_{i}{| }^{0.5}$$

where *x*_*i*_ are the potential outcomes of gamble *x* and *p*(*x*_*i*_) is the rate at which that outcome was produced by the participant in the generation trials. Regression models found that the log-odds of selecting a riskier option increased as its advantage in expected utility increased in both Experiment 1 (*β* = 0.876 [0.535, 1.22], *t*(403) = 5.05, *p* < 0.001) and 2 (*β* = 0.553 [0.255, 0.851], *t*(360) = 3.65, *p* < 0.001). In functional terms, this means that an increase of 1 in the expected utility of gamble B within the current dataset leads to a mean increase in the probability of its selection of 10.9% [6.97%, 14.5%] in Experiment 1 and 11.4% [5.40%, 16.8%] in Experiment 2.

A further question that may be asked is whether generations offer additional predictive ability beyond that given by the initial gamble descriptions. To investigate this, we extended the previous expected utility regression models to include equivalent measures taken purely from gamble descriptions (that is, weighting utilities by listed probabilities rather than generation rates), as well as random slopes for this factor. Including these measures thus controls for the influence of the described information, isolating the specific contribution of generations. These models found that preference for the riskier option again increased as its advantage in generated expected utility increased even when considering described measures in both Experiment 1 (*β* = 0.782 [0.437, 1.127], *t*(402) = 4.46, *p* < 0.001) and 2 (*β* = 0.427 [0.109, 0.745], *t*(359) = 2.64, *p* = 0.009), suggesting this predictive ability goes beyond that provided by the gamble descriptions. This can also be seen by comparing these models’ accuracy in predicting actual selections when either considering or disregarding generated outcomes: predictive accuracy rises when considering the mean generated utility compared to descriptions alone in both Experiment 1 (72.1% vs 66.9%) and 2 (80.4% vs 77.5%), though the size of the increase is fairly small.

This suggests that the outcomes generated by a participant are related to their subsequent choices, though there may be a subjective transformation of these values to psychological utilities. Moreover, this is distinct from the influence of described information, likely reflecting the biases away from objective probabilities in the generated sequences outlined above.

## Discussion

Across two experiments, we find consistent evidence of particular biases in the values generated by decision makers before a choice: both tasks show over-generation of rare events and under-generation of common events overall, but not in initial responses, and low rates of repetition but a preference for movements to more similar values when available. Interestingly, we do not find evidence of bias based on outcome value in either experiment, though common negative correlations between value and probability^43^ may mean over-representation of rare events often additionally causes over-representation of extreme outcomes.

These findings correspond with other studies of random generation outside decision making: sequential dependencies and biases towards uniform weighting have also been observed in generations from both simple artificial^23,28^ and well-established empirical distributions^29,31^. Similar patterns have also been found beyond random generation in other tasks where participants provide long series of responses: for example, repeated estimates of future prices and time intervals show evidence of autocorrelations and excessive variation in responses^44,45^. This may then indicate a common sampling process underlying multiple tasks, impacting not just choice but also judgments and estimates. Such a suggestion forms the basis of the recently proposed Autocorrelated Bayesian Sampler^14^, which uses a consistent mental sampling process for multiple response types. A key advantage of this approach is that it predicts not just common effects across tasks but also connections between them: the same samples can be used to produce multiple responses, meaning biases in one will likely be reflected in another. Investigating these associations may then be a fruitful path for continuation of the present study, for example, following generations with valuations of prospects or confidence in selections.

Our results also illustrate the relation between generation and choice: participants favoured options with higher average utility across their produced sequences, and this effect is distinct from the influence of described information. Such findings imply that generations do offer additional insight into choice processes, potentially indicating the information that may be considered before a decision, and therefore resulting preferences. We should reiterate that this does not mean that explicit generations exactly capture the mental samples used to direct selections, but rather that these responses may be reflective of the output of the mental sampling algorithm, leading to similar if not identical decisions. Indeed, sampling itself is inherently stochastic, potentially producing different sequences and therefore different behaviour if repeated, placing some natural restrictions on predictive ability. The link between generations and choice is also illustrated in the differences between pre- and post-generation decisions observed in Experiment 2, suggesting that extended explicit generation could alter preferences by encouraging greater exploration of the outcome space. At the same time, however, this difference could lead to concerns that explicit generations reflect distinct choice processes from those supporting decisions without generation, potentially challenging our assumption that these tasks can trace the implicit sampling process. This being said, such a suggestion cannot be fully accepted without direct comparisons against control conditions contrasting repeated choices without generations: while we do find some evidence towards this in a reanalysis of existing data, concerns remain given differences in participant samples and task designs. Further tests are thus needed to confirm whether this difference is a reliable result. The present analyses also restrict attention to relatively simple heuristic models which can be easily applied to generated sequences; fitting of more complex models using more extensive choice sets is thus a key avenue for future work.

Such findings offer important implications for mental sampling theories of decision making: by suggesting how information comes to mind ahead of a choice, these generation patterns offer assessments of the plausibility of previously proposed sampling mechanisms. In particular, these results point away from the use of objective probabilities to select samples as in base DFT^1^, but rather a conservative bias similar to that used in its noisier variant DFT_*e*_^11^ and certain parts of the BEAST model^3^. Relatedly, we do not find support for UWS^4^ as generations showed no evidence of an influence of outcome value, though it remains possible that the range of values used in these gambles does not offer sufficient extremes to fully elicit such biases. Our results also argue for consideration of sequential dependencies in generated evidence, as in Query Theory^5^ rather than independent sampling, as well as distinct effects in initial samples not used in any of the noted models. This is not to say the current data entirely refutes any of these models or challenges their ability to predict behaviour; rather, our findings point towards which mechanisms better reflect the availability of choice outcomes in the mind, and should thus be considered in future models seeking to emulate human decision making. These implications do, however, rest on the assumption that the patterns observed in explicit generation are reflective of those underlying mental sampling: if these systems are distinct from one another, then the current findings may be less useful in evaluating these theories. We revisit this point further in the Limitations section below.

Our study also indicates the value of explicit generation tasks in broader explorations of decision making: while the present tasks focus on risky choice given its prevalence in this field, the flexibility of generation allows applications to other forms of decisions, including choices based on experience or between options with multiple attributes. Generations can also offer insight into people’s representations of key distributions^28,30,32^, which could be applied to decision-related aspects like price or quality. More generally, generation tasks are intuitive and easily implemented at relatively low cost, and offer a large amount of data per trial in return.

### Description vs experience

The tasks used here focus on decisions from description, where the distribution of potential outcomes is given in advance, in contrast to decisions from experience, where distributions must be learned through observed outcomes. As noted in the Methods above, this was a deliberate design choice to reduce the influence of memory effects in our results to focus on biases within the generation process itself: descriptions ensure the distribution is complete and unambiguous, meaning any discrepancies can be more concretely attributed to the underlying generative algorithm. This is especially important given that many of the mental sampling models outlined above propose decision makers do sample from descriptions in such tasks^1,3,4,7^; our tasks thus operate at the same level as these models, providing a more direct examination of their assumed sampling patterns. In this sense, our results demonstrate that biases can arise directly from the sampling system even without constraints on memory or learning. This being said, it is also important to consider how the patterns observed here might differ in decisions from experience: are similar deviations from objective probabilities and independent sampling found when gambles are presented through observation? In fact, previous research on the so-called ‘description-experience gap’ might suggest differences should be expected: choice behaviour has been found to systematically differ between these settings, with particular differences in the apparent weighting of rare events (for a review, see Wulff et al.^46^). If such choices are based on mental sampling, then this gap could then be based on distinct sampling behaviour between description and experience, which could be investigated via explicit generations. Contrasts of the current results with those of generation from experience are thus an important future direction to test the generality of these findings.

This links to a related question regarding the process of mental sampling in decisions from description: while sampling from experience can rely on retrieval of events from memory, how are samples drawn when deciding purely from written summaries? Such a question applies more directly to the mechanisms proposed by existing sampling models, which as noted in the Introduction differ between theories: for example, models such as UWS view sampling as mental simulation of choice outcomes^4^, whereas DFT and some versions of the DDM present this as stochastic allocation of attention^1,8^, while DBS suggests memory is in fact used in such cases as well, contrasting described values with past experiences in other settings^2^. The present generation tasks fall closer to the mental simulation reading, directing participants to imagine possible outcomes as if gambles were repeatedly played out, though it should be noted that these experiments were not intended to distinguish these explanations: these tasks aimed to illustrate the properties of the sampling algorithm, but not its supporting mechanisms. Alternative approaches will therefore be needed to separate these depictions, for example, using differing framings to elicit responses.

### Limitations

The most pressing limitation of this study is uncertainty regarding the link between generations and mental sampling: it remains possible that the explicit generations collected in these tasks do not reflect the implicit evidence considered by decision makers when choosing solely from descriptions. Indeed, as noted above, this is partially implied by the differences in choice before and after generations observed in Experiment 2, suggesting explicit generations may alter behaviour and so reflect different underlying processes. This could lead to concerns about the generality of these findings: while generations do appear to be related to subsequent decisions, the observed patterns may not apply to other choices where samples are not explicitly produced. Such decisions could then be based on different sampling processes from those displayed in explicit generations, or perhaps even arise from non-sampling mechanisms. If so, this would heavily restrict the implications of these findings for the choice models described above. This is, unfortunately, a difficult issue to resolve: as noted in the Introduction, mental samples are inherently unobservable and so can only be inferred with secondary measures, inevitably introducing some degree of uncertainty. It may, however, be possible to further examine this connection by cross-referencing generation with other process tracing methods, such as eye-tracking^8,18,19^, to determine whether these provide consistent patterns.

Such a distinction between explicit generation tasks and mental sampling theories would be slightly surprising given the close resemblance between these processes: if decision makers sample outcomes internally before a choice, why should asking them to externalise this process alter behaviour? One possibility is that the generation phase used here is simply much longer than the time usually taken for such decisions (2 min vs  ~ 10 s in Erev et al.^3^): our participants may then be considering a larger set of samples than they otherwise would, whereas decisions from descriptions alone might rely on only a few initial samples. This is particularly relevant given the apparent differences between initial and subsequent samples suggested by these data: smaller sample counts may then display different patterns than extended sampling, leading to different preferences. Such extended generation is, however, important to separate biases within the sampling process from effects arising simply from small sample sizes; this being said, variants of this task with differing generation durations or allowing participants to stop generations themselves could offer valuable contrasts with the current findings. Alternatively, sampling could differ when actively comparing two alternatives rather than simply generating from each option (for example, focusing on pairs of outcomes with larger differences to aid discrimination^4^), though it is notable that generations in the current tasks were made in a comparative context in advance of a known forthcoming choice. Further comparisons of choice both with and without explicit generation are therefore needed to confirm whether this is a robust difference.

Differences could also arise from alternate readings of the task by the participants, leading to distinct sampling behaviour from that used for choice. For example, participants may have sought to use their generations to communicate the gambles’ distribution of outcomes, with the observed biases resulting from restrictions on such communication in the task: within a limited sample size, rare events may need to be overrepresented to prevent these being completely omitted, and repetitions avoided to reduce redundant information. It should, however, be noted that our task made no reference to communication of the gambles, so this reading was certainly not encouraged, but could perhaps have arisen as a default approach (though this itself is a strong claim). Alternatively, participants may have been trying to emulate truly random behaviour rather than following their internal sampling process. The observed biases may then reflect an inability to accurately emulate randomness, as observed in previous random generation tasks^21,23,29^. This reading is more in line with our framing of the task as repeated imagined draws, though this may not in fact alter behaviour: a recent comparison found consistent patterns when generation tasks were framed either as random generation or reporting outcomes as they came to mind^47^.

If these alternate readings were used, then the current findings may be less helpful for evaluating choice models as they reflect different motivations, and therefore different behavioural patterns. At the same time, these data would continue to illustrate the broader accessibility of potential outcomes in the mind, which could still inadvertently influence various judgments regarding the gambles: even if samples are not intentionally selected to guide choice, the set of evidence collected might impact perceptions of the value of the target or expectations of actual draws. This is again suggested by the apparent shift in preferences before and after generations in Experiment 2. Following generations with alternate responses may then be valuable to test for such influences. This also raises a question of how many sampling methods people propose to have: do we hold distinct sampling algorithms for any specific task we may face, or is there a common system applied across tasks? If patterns are consistent between tasks, this may be more parsimoniously explained by a single underlying process than separate specialised systems. It may therefore be valuable to contrast the present task with variants explicitly encouraging these interpretations to test for any differences in behaviour.

There are also more basic methodological aspects of the current elicitation method which could play a role in these findings: for example, some biases might simply be due to participants misunderstanding the reward function, incorrectly believing that focusing on producing the rarer large gains increases their potential bonus at the end of the task. Participants may also have engaged in strategic rather than truly random responding, for example, cycling through outcomes, thus leading to fewer repetitions and more alternations. Contrasts with other elicitation methods, such as verbal or graphical responses, as well as alternative instructions on the generation procedure, are thus advisable to examine the impact of this factor.

### Conclusion

Explicit generation tasks provide a simple method to trace the processes underlying decision-making, potentially revealing key biases in considered information. While these methods have their limitations, our results demonstrate the promise of this approach, and provide a foundation for further investigation of human decision systems.

## Supplementary information

Transparent Peer Review file

Supplementary Information

## Data Availability

All empirical data collected for this study are available on the Open Science Framework at: https://osf.io/f5wje/.

## Code availability

Code used in the described analyses are available on the Open Science Framework at: https://osf.io/f5wje/(10.17605/OSF.IO/F5WJE).

## References

[1] Busemeyer, J. R. & Townsend, J. T. Decision field theory: a dynamic-cognitive approach to decision making in an uncertain environment. *Psychol. Rev.* **100**, 432 (1993). 8356185 10.1037/0033-295x.100.3.432

[2] Stewart, N., Chater, N. & Brown, G. D. Decision by sampling. *Cogn. Psychol.* **53**, 1–26 (2006). 16438947 10.1016/j.cogpsych.2005.10.003

[3] Erev, I., Ert, E., Plonsky, O., Cohen, D. & Cohen, O. From anomalies to forecasts: toward a descriptive model of decisions under risk, under ambiguity, and from experience. *Psychol. Rev.* **124**, 369 (2017). 28277716 10.1037/rev0000062

[4] Lieder, F., Griffiths, T. L. & Hsu, M. Overrepresentation of extreme events in decision making reflects rational use of cognitive resources. *Psychol. Rev.* **125**, 1–32 (2018). 29035078 10.1037/rev0000074 PMC5773401

[5] Johnson, E. J., Häubl, G. & Keinan, A. Aspects of endowment: a query theory of value construction. *J. Exp. Psychol. Learn. Mem. Cogn.* **33**, 461 (2007). 17470000 10.1037/0278-7393.33.3.461

[6] Ratcliff, R. A theory of memory retrieval. *Psychol. Rev.* **85**, 59–108 (1978).

[7] Ratcliff, R., Smith, P. L., Brown, S. D. & McKoon, G. Diffusion decision model: current issues and history. *Trends Cogn. Sci.* **20**, 260–281 (2016). 26952739 10.1016/j.tics.2016.01.007 PMC4928591

[8] Krajbich, I., Armel, C. & Rangel, A. Visual fixations and the computation and comparison of value in simple choice. *Nat. Neurosci.* **13**, 1292–1298 (2010). 20835253 10.1038/nn.2635

[9] Kellen, D., Steiner, M. D., Davis-Stober, C. P. & Pappas, N. R. Modeling choice paradoxes under risk: from prospect theories to sampling-based accounts. *Cogn. Psychol.* **118**, 101258 (2020). 32058123 10.1016/j.cogpsych.2019.101258

[10] Zhao, W. J., Walasek, L. & Bhatia, S. Psychological mechanisms of loss aversion: a drift-diffusion decomposition. *Cogn. Psychol.* **123**, 101331 (2020). 32777328 10.1016/j.cogpsych.2020.101331

[11] Bhatia, S. Sequential sampling and paradoxes of risky choice. *Psychon. Bull. Rev.* **21**, 1095–1111 (2014). 24898202 10.3758/s13423-014-0650-1

[12] Lieder, F., Griffiths, T. L., Huys, Q. J. & Goodman, N. D. The anchoring bias reflects rational use of cognitive resources. *Psychon. Bull. Rev.* **25**, 322–349 (2018). 28484952 10.3758/s13423-017-1286-8

[13] Spicer, J., Zhu, J.-Q., Chater, N. & Sanborn, A. N. Perceptual and cognitive judgments show both anchoring and repulsion. *Psychol. Sci.* **33**, 1395–1407 (2022). 35876741 10.1177/09567976221089599

[14] Zhu, J.-Q., Sundh, J., Spicer, J., Chater, N. & Sanborn, A. N. The Autocorrelated Bayesian sampler: a rational process for probability judgments, estimates, confidence intervals, choices, confidence judgments, and response times. *Psychol. Rev.* **131**, 456–493 (2024). 37289507 10.1037/rev0000427 PMC11115360

[15] Birnbaum, M. H. New paradoxes of risky decision making. *Psychol. Rev.* **115**, 463 (2008). 18426300 10.1037/0033-295X.115.2.463

[16] Vanunu, Y., Hotaling, J. M. & Newell, B. R. Elucidating the differential impact of extreme-outcomes in perceptual and preferential choice. *Cogn. Psychol.* **119**, 101274 (2020). 32062088 10.1016/j.cogpsych.2020.101274

[17] Zhao, W. J., Richie, R. & Bhatia, S. Process and content in decisions from memory. *Psychol. Rev.* **129**, 73–106 (2022). 34472948 10.1037/rev0000318

[18] Stewart, N., Gächter, S., Noguchi, T. & Mullett, T. L. Eye movements in strategic choice. *J. Behav. Decis. Mak.* **29**, 137–156 (2016). 27513881 10.1002/bdm.1901 PMC4959529

[19] Pachur, T., Schulte-Mecklenbeck, M., Murphy, R. O. & Hertwig, R. Prospect theory reflects selective allocation of attention. *J. Exp. Psychol. Gen.* **147**, 147 (2018). 29369680 10.1037/xge0000406

[20] Wagenaar, W. A. Generation of random sequences by human subjects: a critical survey of literature. *Psychol. Bull.* **77**, 65 (1972).

[21] Nickerson, R. S. The production and perception of randomness. *Psychol. Rev.* **109**, 330 (2002). 11990321 10.1037/0033-295x.109.2.330

[22] Oskarsson, A. T., Van Boven, L., McClelland, G. H. & Hastie, R. What’s next? judging sequences of binary events. *Psychol. Bull.* **135**, 262 (2009). 19254080 10.1037/a0014821

[23] Towse, J. N. On random generation and the central executive of working memory. *Br. J. Psychol.* **89**, 77–101 (1998). 9532724 10.1111/j.2044-8295.1998.tb02674.x

[24] Schulz, M.-A., Schmalbach, B., Brugger, P. & Witt, K. Analysing humanly generated random number sequences: a pattern-based approach. *PLoS One* **7**, e41531 (2012). 22844490 10.1371/journal.pone.0041531 PMC3402418

[25] Biesaga, M., Talaga, S. & Nowak, A. The effect of context and individual differences in human-generated randomness. *Cogn. Sci.* **45**, e13072 (2021). 34913501 10.1111/cogs.13072 PMC9285827

[26] Camerer, C. *Behavioral Game Theory: Experiments in Strategic Interaction* (Princeton University Press, 2003).

[27] Osborne, M. J. *An Introduction to Game Theory* (Oxford University Press, 2004).

[28] Li, Y.-X. et al. Does outcome utility bias the mental simulations of risky events? Preprint at *PsyArXiv*10.31234/osf.io/awdr7_v2 (2026).

[29] Castillo, L., León-Villagrá, P., Chater, N. & Sanborn, A. N. Explaining the flaws in human random generation as local sampling with momentum. *PLoS Comput. Biol.* **20**, e1011739 (2024). 38181041 10.1371/journal.pcbi.1011739 PMC10796055

[30] León-Villagrá, P., Castillo, L., Falbén, J., Chater, N. & Sanborn, A. N. Eliciting beliefs with random generation tasks. Preprint at 10.31234/osf.io/687au (2024).

[31] Falbén, J. K., Castillo, L., León-Villagrá, P., Chater, N. & Sanborn, A. N. Biased mind or biased world? Assessing the accuracy of cultural beliefs that underlie social judgments. Preprint at *PsyArXiv*10.31234/osf.io/5hdbp (2024).

[32] Ronayne, D. & Brown, G. D. Multi-attribute decision by sampling: An account of the attraction, compromise and similarity effects. *J. Math. Psychol.* **81**, 11–27 (2017).

[33] Zhu, J.-Q., León-Villagrá, P., Chater, N. & Sanborn, A. N. Understanding the structure of cognitive noise. *PLoS Comput. Biol.* **18**, e1010312 (2022). 35976980 10.1371/journal.pcbi.1010312 PMC9423631

[34] Faul, F., Erdfelder, E., Buchner, A. & Lang, A.-G. Statistical power analyses using g* power 3.1: tests for correlation and regression analyses. *Behav. Res. Methods* **41**, 1149–1160 (2009). 19897823 10.3758/BRM.41.4.1149

[35] Tversky, A. & Kahneman, D. Advances in prospect theory: Cumulative representation of uncertainty. *J. Risk Uncertain.* **5**, 297–323 (1992).

[36] Brandstätter, E., Gigerenzer, G. & Hertwig, R. The priority heuristic: making choices without trade-offs. *Psychol. Rev.* **113**, 409–432 (2006). 16637767 10.1037/0033-295X.113.2.409 PMC2891015

[37] Rieskamp, J. The probabilistic nature of preferential choice. *J. Exp. Psychol. Learn. Mem. Cogn.* **34**, 1446–1465 (2008). 18980407 10.1037/a0013646

[38] Glöckner, A. & Pachur, T. Cognitive models of risky choice: parameter stability and predictive accuracy of prospect theory. *Cognition* **123**, 21–32 (2012). 22226615 10.1016/j.cognition.2011.12.002

[39] Spicer, J., Mullett, T. L. & Sanborn, A. N. Repeated risky choices become more consistent with themselves but not expected value, with no effect of matched trial order. *Judgm. Decis. Mak.* **19**, e2 (2024).

[40] Azab, H., Ruskin, D. & Kidd, C. Adults’ guesses on probabilistic tasks reveal incremental representativeness biases. In *Proc. the 38th Annual Meeting of the Cognitive Science Society*, Vol. **38** (University of California, 2016); https://escholarship.org/uc/item/4vp9n4wm

[41] Ljung, G. M. & Box, G. E. On a measure of lack of fit in time series models. *Biometrika* **65**, 297–303 (1978).

[42] Towse, J. N. & Valentine, J. D. Random generation of numbers: a search for underlying processes. *Eur. J. Cogn. Psychol.* **9**, 381–400 (1997).

[43] Pleskac, T. J. & Hertwig, R. Ecologically rational choice and the structure of the environment. *J. Exp. Psychol. Gen.* **143**, 2000–2019 (2014). 24979239 10.1037/xge0000013

[44] Zhu, J.-Q., Spicer, J., Sanborn, A. N. & Chater, N. Cognitive variability matches speculative price dynamics. *Cognition* **250**, 105858 (2024). 38906014 10.1016/j.cognition.2024.105858

[45] Spicer, J., Zhu, J.-Q., Chater, N. & Sanborn, A. N. How do people predict a random walk? Lessons for models of human cognition. *Psychol. Rev.* **131**, 1069–1113 (2024). 39298225 10.1037/rev0000493

[46] Wulff, D. U., Mergenthaler-Canseco, M. & Hertwig, R. A meta-analytic review of two modes of learning and the description-experience gap. *Psychol. Bull.* **144**, 140 (2018). 29239630 10.1037/bul0000115

[47] Castillo, L., León-Villagrá, P., Falbén, J. K., Chater, N. & Sanborn, A. N. Random generation is what comes to mind in naturalistic settings. *Cognition* **274**, 106554 (2026). 10.1016/j.cognition.2026.106554 42092370

