# Supplementary material for: Generated outcomes in risky choice reveal biased sampling and sequential dependencies
Source: Commun Psychol. 2026 May 7;4:104. doi: 10.1038/s44271-026-00467-y (PMC13364634; doi:10.1038/s44271-026-00467-y)
Supplement: Supplementary file 2 — Supplementary Information [file 44271_2026_467_MOESM2_ESM.pdf]

Generated Outcomes in Risky Choice Reveal Biased Sampling and Sequential Dependencies

Jake Spicer, Yun-Xiao Li, Lucas Castillo, Johanna K. Falbén, C. Stella Qian, Adam N. Sanborn

Supplementary Information

Supplementary Notes 1

Choice Effect Descriptions

This section gives more detail on the set of choice phenomena evaluated by the gamble set used in our experiments, expanding on the brief summary in Table 1 of the main text. The 8 gamble pairs test 6 specific effects via particular choice patterns either within a gamble pair or between two pairs.

Certainty Effect

The certainty effect reflects a preference for positive outcomes that are certain to occur (i.e., probability 1) over risky alternatives even when these may have lower expected value. Within this gamble set, this is evaluated by comparing choice rates between Pairs 1 and 2: because Gamble A offers a certain outcome in Pair 1, A is preferred; however, when probabilities are scaled down by a constant factor of 0.25 in Pair 2, preference switches to Gamble B.

Reflection

The reflection effect captures an inversion of the certainty effect for losses: in this case, a chance of a loss is preferred to a certain loss. This is tested by Pair 3: decision makers prefer Gamble B as it offers the possibility of avoiding the certain loss in Gamble A.

Overweighting of Rare Events

Decision makers often seem to overweight rare events in decisions from description, preferring options offering low probabilities of large gains (or avoiding options with low probabilities of large losses). This is tested here by Pair 4: while both gambles have equal expected value, decision makers tend to prefer Gamble B which offers a small chance of a large win.

Loss Aversion

Loss aversion describes a tendency to overweight losses in decision making, seemingly treating negative values as more impactful than gains of equal magnitude. This is tested here by Pair 5: an equal chance of gains and losses of equal magnitude is less attractive than a certainty of no change despite equal expected value.

Risk Aversion

Risk aversion describes a tendency to prefer safer over riskier options even when riskier options offer higher expected values. This is tested here by Pair 6, which reflects a closed form of the St. Petersburg problem: decision makers prefer a certain smaller gain over a gamble offering increasingly small probabilities of increasingly larger values.

Splitting

Splitting reflects a change in preference when outcome probabilities are split into smaller components: splitting a gain into several smaller possibilities makes that option more attractive even when it reduces expected value. This is tested by Pairs 7 and 8: Gamble B in Pair 8 divides the highest outcome of Gamble B in Pair 7 into three lower probability and lower value components, but this results in an increased preference for the riskier option.

Supplementary Notes 2

Over-generation of Extreme Outcomes

While the regressions in the main analyses suggest no significant influence of outcome value on generation, negative correlations between value and probability in the gamble set could mean that more extreme outcomes may still be over-represented in participants' sequences via the bias in probability. To investigate this possibility, we ran a supplemental analysis examining generation rates according to value after the true probability has been removed by taking the deviation between each outcome's generated rate and its true probability. These deviations were then regressed on outcome value for each experiment.

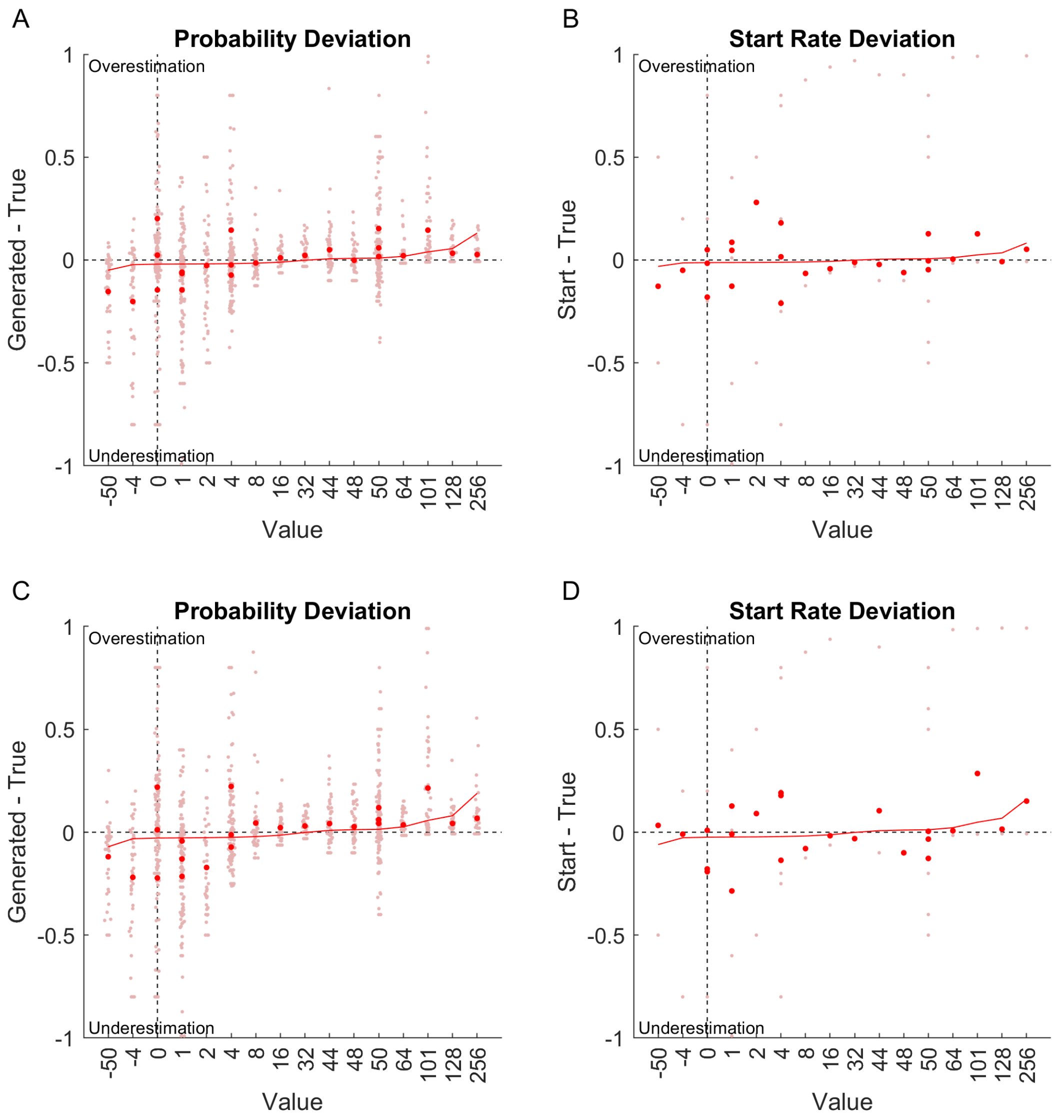

Figure S1

Generation Rates by Outcome Value

Deviation in overall generation rate (left column) and start rate (right column) from true probability in Experiments 1 (top row) and 2 (bottom row). Light points represent individual-level data and bold points represent means across participants (n = 51 participants in Experiment 1 and n = 46 participants in Experiment 2). Red lines represent regression fits across individual data (n = 1218 rates in Experiment 1; n = 1088 rates in Experiment 2), while dotted lines mark zero.

Results from these analyses are shown in Figure S1. In both tasks, deviation was found to increase with outcome value (Experiment 1: $\beta = 0.0006$ [0.0004, 0.0008], $t(1216) = 5.58$,

$p < .001$; Experiment 2: β = 0.0008 [0.0005, 0.0012], $t(1086) = 5.21$, $p < .001$), with higher value outcomes more likely to be over-generated and lower outcomes under-generated. This test does however overlook the relative value of an outcome within a choice pair (for example, £4 is the best outcome in Pair 1 but relatively low in Pair 6). As such, we performed an alternate version of this test to account for differences in context by converting each outcome into a deviation from the expected value (EV) of that gamble. This found similar results to the previous tests, with higher deviations being more likely to be over-generated, (Experiment 1: β = 0.0006 [0.0004, 0.0008], $t(1216) = 5.58$, $p < .001$; Experiment 2: β = 0.0009 [0.0005, 0.0012], $t(1086) = 5.18$, $p < .001$).

We also performed a version of this analysis for the starting points, in this case using deviation between start rate and true probability. Mixed-model regressions again found a positive relationship between value and deviation in both tasks (Experiment 1: β = 0.0004 [0, 0.0007], $t(1208) = 2.08$, $p = .038$; Experiment 2: β = 0.0007 [0.0002, 0.0012], $t(1062) = 2.80$, $p = .005$). This was also replicated when adjusting values according to the EV of that gamble (Experiment 1: β = 0.0004 [0, 0.0007], $t(1208) = 2.08$, $p = .037$; Experiment 2: β = 0.0007 [0.0002, 0.0013], $t(1062) = 2.80$, $p = .005$).

These results illustrate that while generations may be primarily driven by the stated probabilities, this can cause biases in considered evidence towards extreme value outcomes. This predominantly focuses on large gains here as there are few extreme losses in the current gamble set; further contrasts with differing choice sets are thus needed to determine whether this finding crosses domains or is sign-dependent.

Supplementary Notes 3

Autocorrelation Function Comparison Results

This section gives full results for the comparisons between the empirical autocorrelation function in participants' generated sequences and that expected from equivalent independent series. Table S1 gives results for Experiment 1, and Table S2 gives results for Experiment 2. In

both cases, no significant differences between the observed and simulated series were found at any lag.

Lag	ACF	IID	β	t	p
1	-0.101 (-0.136, -0.066)	-0.050 (-0.053, -0.046)	-0.035 (-0.091, 0.022)	-1.20	.228
2	-0.039 (-0.066, -0.012)	-0.045 (-0.048, -0.043)	0.012 (-0.044, 0.067)	0.42	.677
3	-0.069 (-0.092, -0.046)	-0.042 (-0.043, -0.040)	-0.023 (-0.083, 0.037)	-0.76	.450
4	-0.017 (-0.042, 0.008)	-0.039 (-0.04, -0.037)	0.022 (-0.043, 0.087)	0.67	.504
5	-0.006 (-0.032, 0.020)	-0.036 (-0.037, -0.034)	0.031 (-0.035, 0.098)	0.92	.359
6	-0.033 (-0.056, -0.010)	-0.032 (-0.033, -0.031)	0.001 (-0.060, 0.062)	0.04	.966
7	-0.024 (-0.044, -0.004)	-0.030 (-0.031, -0.029)	0.006 (-0.057, 0.070)	0.20	.841
8	-0.014 (-0.033, 0.005)	-0.028 (-0.028, -0.027)	0.013 (-0.049, 0.074)	0.40	.686
9	-0.022 (-0.040, -0.004)	-0.025 (-0.026, -0.024)	0.003 (-0.059, 0.064)	0.09	.927
10	0.015 (-0.005, 0.035)	-0.023 (-0.023, -0.022)	0.035 (-0.027, 0.098)	1.11	.269

Table S1

Autocorrelation function results from Experiment 1. ACF gives the mean autocorrelation function across participants and gambles, while IID gives the expected value assuming independence. β, *t and p give results from the regression on the differences between observed and expected values (df = 3457). Brackets give 95% confidence intervals.*

Supplementary Notes 4

Choice Rule Analysis

Choice Rule Definitions

We here provide further detail on the choice rules considered in our main analyses. Note that while these rules have previously been applied to objective gamble features listed in their descriptions[1,2], in our analyses objective probabilities are replaced with the rate at which each outcome was generated in our experiments.

Lag	ACF	IID	β	*t*	*p*
1	-0.102 (-0.141, -0.063)	-0.054 (-0.058, -0.051)	-0.042 (-0.104, 0.019)	-1.35	.177
2	-0.012 (-0.043, 0.020)	-0.050 (-0.052, -0.047)	0.042 (-0.026, 0.111)	1.21	.227
3	-0.062 (-0.089, -0.036)	-0.046 (-0.048, -0.043)	-0.020 (-0.082, 0.042)	-0.63	.529
4	-0.010 (-0.037, 0.016)	-0.041 (-0.043, -0.040)	0.035 (-0.043, 0.112)	0.88	.380
5	-0.034 (-0.059, -0.008)	-0.037 (-0.039, -0.036)	0.000 (-0.066, 0.066)	-0.01	.993
6	-0.019 (-0.044, 0.005)	-0.034 (-0.035, -0.033)	0.016 (-0.059, 0.091)	0.41	.684
7	-0.039 (-0.061, -0.017)	-0.030 (-0.031, -0.029)	-0.013 (-0.087, 0.060)	-0.36	.720
8	-0.018 (-0.041, 0.005)	-0.027 (-0.027, -0.026)	0.011 (-0.067, 0.088)	0.27	.788
9	-0.034 (-0.055, -0.013)	-0.025 (-0.025, -0.024)	-0.011 (-0.081, 0.059)	-0.32	.753
10	0.007 (-0.014, 0.028)	-0.023 (-0.023, -0.022)	0.029 (-0.047, 0.106)	0.76	.448

Table S2

Autocorrelation function results from Experiment 2. ACF gives the mean autocorrelation function across participants and gambles, while IID gives the expected value assuming independence. β, t and p give results from the regression on the differences between observed and expected values (df = 3140). Brackets give 95% confidence intervals.

Higher Mean

This rule simply compares the mean outcome across generations from each option, essentially reflecting the subjective expected value of the alternatives.

Expected Utility

Expected utility reflects a common assumption in previous decision making research in which objective values from gamble descriptions are converted to subjective utilities[3–5]. As noted in the main text, we used a basic concave utility function via a power law with exponent 0.5:

$$E[U(x)] = \sum_i p(x_i) sign(x_i) |x_i|^{0.5} \tag{1}$$

where x_i are the potential outcomes of gamble x and $p(x_i)$ is the rate at which that outcome was produced by the participant in the generation trials. This is then similar to the Higher Mean rule above but aggregating subjective rather than objective values.

Probable

The Probable rule classifies outcomes as either 'probable' or 'improbable' based on whether their generated rate across the sequence was greater or less than the uniform probability for the number of outcomes in that gamble. Each alternative is then valued according to the mean of the 'probable' outcomes only.

Least Likely

The Least Likely rule compares the generated rates of the respective lowest outcome of each gamble, assessing which option is less likely to pay out its minimum.

Most Likely

The Most Likely rule compares the most commonly generated outcomes of each gamble, assessing which has the better value.

Lexicographic

The Lexicographic rule extends the Most Likely rule in case no difference is found between the most commonly generated outcomes; in this case, comparisons are then made between the second most common outcomes of each gamble, and so on until a difference is found.

Tallying

The Tallying heuristic predicts preference using a set of four comparisons between alternatives: which has the higher minimum outcome, the higher maximum outcome, a lower probability of the minimum outcome, and a higher probability of the maximum outcome. Tallying then counts the number of comparisons won by each option and determines which holds the most wins.

Equiprobable

The Equiprobable rule compares the mean outcome from each option ignoring their generated rates, essentially assuming these were equally represented.

Equal Weight

The Equal Weight rule compares the sum of the unique outcomes generated for each option, also ignoring the exact rates at which these were generated.

Better-than-Average

The Better-than-Average rule compares the number of outcomes from each gamble that are higher than the grand average of all outcomes across both gambles, again ignoring their generated rates.

Minimax

The Minimax rule compares the minimum outcome generated for each option without considering their exact generated rates.

Maximax

The Maximax rule compares the maximum generated outcome from each option, again without considering their generated rates.

Choice Regression Results

This section provides further results for the mixed-model regressions performed for each choice rule. Tables S3 and S4 give intercepts and coefficients for all models from Experiment 1 and 2 respectively. As the scales used by each rule differ, we standardised each difference measure (i.e., mean = 0, sd = 1) to aid comparison of coefficients between rules, meaning resulting estimates all reflect predicted change in the log-odds of selecting the riskier option from an increase of 1 standard deviation in the respective measure.

Pre-Generation Choice

As a supplementary test, we also used generated sequences to predict choices in the pre-generation choice block of Experiment 2. Results are summarised in Table S5: in this case,

Rule	Intercept	Coefficient	*t*	*p*
Higher Mean	-0.152 (-0.417, 0.113)	0.634 (0.345, 0.923)	4.31	<.001***
Expected Utility	-0.140 (-0.419, 0.139)	0.876 (0.535, 1.217)	5.05	<.001***
Probable	-0.201 (-0.450, 0.048)	0.523 (0.249, 0.797)	3.75	<.001***
Least Likely	-0.190 (-0.438, 0.058)	-0.109 (-0.315, 0.097)	-1.04	.299
Most Likely	-0.204 (-0.453, 0.044)	0.490 (0.227, 0.753)	3.66	<.001***
Lexicographic	-0.205 (-0.454, 0.043)	0.493 (0.231, 0.755)	3.70	<.001***
Tallying	-0.208 (-0.464, 0.049)	0.361 (0.123, 0.599)	2.98	.003**
Equiprobable	-0.182 (-0.434, 0.070)	0.313 (0.098, 0.527)	2.87	.004**
Equal Weight	-0.188 (-0.438, 0.062)	-0.020 (-0.258, 0.218)	-0.17	.868
Better-than-Average	-0.178 (-0.428, 0.073)	0.008 (-0.215, 0.231)	0.07	.945
Minimax	-0.190 (-0.438, 0.058)	0.197 (-0.012, 0.406)	1.85	.064
Maximax	-0.184 (-0.431, 0.063)	0.130 (-0.085, 0.346)	1.19	.236

Table S3

Choice rule regression results from Experiment 1. Coefficients provide predicted change in log-odds for an increase of 1 standard deviation in the rule measure, while t and p test whether this significantly differs from zero ($df = 403$). Asterisks denote significant predictors, while brackets give 95% confidence intervals.

the Better-than-Average rule provided the best mapping between generations and choice, though the Equal Weight rule was not substantially worse ($LR = 0.376$). Subsequent regressions however found no significant effect of the key measure taken from generations for either the Equal Weight ($\beta = 0.101$ [-0.358, 0.561], $t(359) = 0.43$, $p = .665$) or Better-than-Average ($\beta = -0.111$ [-0.506, 0.283], $t(359) = 0.55$, $p = .580$) rules when including equivalent measures from gamble descriptions. This suggests that while these are the best mappings within the rule set, their effect is not reliable after controlling for the influence of described information. In addition, the

Rule	Intercept	Coefficient	t	p
Higher Mean	0.082<br>(-0.256, 0.420)	0.364<br>(0.021, 0.707)	2.09	.037*
Expected Utility	0.081<br>(-0.263, 0.426)	0.553<br>(0.255, 0.851)	3.65	<.001***
Probable	0.050<br>(-0.274, 0.373)	0.223<br>(-0.029, 0.474)	1.74	.082
Least Likely	0.053<br>(-0.279, 0.386)	-0.194<br>(-0.425, 0.036)	-1.66	.098
Most Likely	0.055<br>(-0.267, 0.377)	0.110<br>(-0.152, 0.373)	0.83	.408
Lexicographic	0.063<br>(-0.255, 0.382)	0.181<br>(-0.120, 0.481)	1.18	.238
Tallying	0.066<br>(-0.254, 0.386)	0.083<br>(-0.202, 0.369)	0.57	.567
Equiprobable	0.061<br>(-0.274, 0.396)	0.172<br>(-0.102, 0.447)	1.24	.217
Equal Weight	0.024<br>(-0.325, 0.374)	-0.155<br>(-0.486, 0.176)	-0.92	.357
Better-than-Average	0.049<br>(-0.282, 0.381)	0.055<br>(-0.213, 0.322)	0.40	.687
Minimax	0.047<br>(-0.277, 0.371)	0.015<br>(-0.218, 0.248)	0.13	.897
Maximax	0.046<br>(-0.287, 0.380)	0.048<br>(-0.220, 0.316)	0.35	.724

Table S4

Choice rule regression results from Experiment 2. Coefficients provide predicted change in log-odds for an increase of 1 standard deviation in the rule measure, while t and p test whether this significantly differs from zero ($df = 360$). Asterisks denote significant predictors, while brackets give 95% confidence intervals.

likelihood of all rules is substantially lower for the pre-generation choice block, implying measures taken from generations offer worse fits to these data. This may not be surprising however given that these choices preceded generations, and so are unlikely to have been influenced by them, though these may have been based on a similar sampling process.

Rule	Log Likelihood	*LR*
Higher Mean	-223.43	<0.001
Expected Utility	-221.19	0.002
Probable	-216.15	0.251
Least Likely	-219.43	0.009
Most Likely	-217.62	0.058
Lexicographic	-220.03	0.005
Tallying	-221.52	0.001
Equiprobable	-218.76	0.019
Equal Weight	-215.75	0.376
Better-than-Average	-214.77	1
Minimax	-217.01	0.107
Maximax	-219.88	0.006

Table S5

Choice rule comparison results for pre-generation choice in Experiment 2. Log likelihoods give fit to empirical data (higher values reflect better fits), while LRs give likelihood ratios between each rule and the best-fitting rule (Better-than-Average).

Supplementary References

1. Brandstätter, E., Gigerenzer, G. & Hertwig, R. The priority heuristic: making choices without trade-offs. *Psychological Review* **113,** 409–432. doi:10.1037/0033-295X.113.2.409 (2006).

2. Spicer, J., Mullett, T. L. & Sanborn, A. N. Repeated risky choices become more consistent with themselves but not expected value, with no effect of matched trial order. *Judgment and Decision Making* **19,** e2. doi:10.1017/jdm.2023.41 (2024).

3. Tversky, A. & Kahneman, D. Advances in prospect theory: Cumulative representation of uncertainty. *Journal of Risk and Uncertainty* **5,** 297–323. doi:10.1007/BF00122574 (1992).

4. Busemeyer, J. R. & Townsend, J. T. Decision field theory: a dynamic-cognitive approach to decision making in an uncertain environment. *Psychological Review* **100,** 432. doi:10.1037/0033-295X.100.3.432 (1993).

5. Bhatia, S. Sequential sampling and paradoxes of risky choice. *Psychonomic Bulletin & Review* **21,** 1095–1111. doi:10.3758/s13423-014-0650-1 (2014).
