# Supplementary material for: Generated outcomes in risky choice reveal biased sampling and sequential dependencies
Source: Commun Psychol. 2026 May 7;4:104. doi: 10.1038/s44271-026-00467-y (PMC13364634; doi:10.1038/s44271-026-00467-y)
Supplement: Supplementary file 1 — Transparent Peer Review file [file 44271_2026_467_MOESM1_ESM.pdf]

nature portfolio

Peer Review File

Generated Outcomes in Risky Choice Reveal Biased Sampling and Sequential Dependencies

Corresponding Author: Dr Jake Spicer

This file contains all editorial decision letters in order by version, followed by all author rebuttals in order by version.

Version 0:

Decision Letter:

** Please ensure you delete the link to your author homepage in this e-mail if you wish to forward it to your coauthors **

Dear Dr Spicer,

Thank you for your patience during the peer-review process. Your manuscript titled "Mental Sampling in Preferential Choice: Tracing the Process using Random Generation" has now been seen by 5 reviewers, whose comments are appended below. You will see that they find your work of some potential interest. However, they have raised quite substantial concerns that must be addressed.

In light of these comments, we cannot accept the manuscript for publication but would be interested in considering a revised version that fully addresses these serious concerns. Please bear in mind that we will be reluctant to approach the reviewers again in the absence of substantial revisions.

If you choose to take up this option, please highlight all changes in the manuscript text file, and provide a detailed point-by-point reply to the reviewers.

Editorially, we consider it important that the revised manuscript addresses the conceptual and methodological concerns of the reviewers through additional description, analysis, and experimentation where needed. Please clarify the rationale for the research question and hypotheses, strengthen or discuss the theoretical assumptions brought up by the reviewers (related to random generation, to mental sampling, and their association), and provide more methodological clarify.

Please ensure you follow our statistical guidelines when reporting statistics (https://www.nature.com/commspsychol/submit/submission-guidelines#statistical-guidelines). Please note in particular our requirements for the reporting and interpretation of null-results. Non-significant findings derived from null-hypotheses significance tests should be reported in full, but may not be interpreted. Where you interpret null results, this interpretation must be based on Bayes Factors or equivalence tests.

If you decide to revise the manuscript in response to these issues, please also implement all requests in the attached Mandatory Revision Requests document. All requirements listed in this document need to be fully met, or the work will be returned to you for further revisions without peer review. This workflow is in place to increase the likelihood that the paper will ultimately be accepted for publication. It reduces the number of rounds of revision (and review) and ensures that the reviewers vet a version of the article that is compliant with journal policies. If you have any questions regarding the required revisions, please contact the journal prior to resubmission to avoid a negative outcome.

If the revision process takes significantly longer than five months, we will be happy to reconsider your paper at a later date, provided it still presents a significant contribution to the literature at that stage.
We would appreciate it if you could keep us informed about an estimated timeline for resubmission, to facilitate our planning.

Please submit the following items:

- Revised manuscript

- Point-by-point response to the referees' comments
- Mandatory Revision Requests Table (attached).
- Cover letter (as a separate document)

Via this link:
Link Redacted

** This url links to your confidential home page and associated information about manuscripts you may have submitted or are reviewing for us. If you wish to forward this email to co-authors, please delete the link to your homepage first **

Thank you for the opportunity to review your work.

Best regards,

Caroline Charpentier

Caroline Charpentier, PhD
Editorial Board Member
Communications Psychology
orcid.org/0000-0002-7283-0738

REVIEWER EXPERTISE:
Reviewer #1 mental sampling, random generation processes
Reviewer #2 mental sampling, risky decision-making
Reviewer #4 risky decision-making

REVIEWER REPORTS:

Reviewer #1 (Remarks to the Author):

In the manuscript, the authors investigate the sampling process in the mental sampling mechanism. In two studies, they asked participants to generate elements from 8 different distributions and compare their results against series generated at random. Their results showed over-generation of rare elements while under-generation of common outcomes. Moreover, they showed the avoidance of the direct repetition of elements. Their finding suggest that the process of mental sampling is not driven by a random process.

In general, the paper is difficult to follow. While the authors try to follow Springer's structure of the paper by having the detailed description of the methods after the results, it makes the paper hard to comprehend. For example, the brief introduction of the procedure before the results, in my opinion, is not enough to understand exactly the task participants had to follow. I would suggest having a more traditional presentation of the method section with a detailed description of the procedure before the results (as far as I know, it is not mandatory to follow this one).

When it comes to the structure of the paper, I would suggest expanding the introduction. So far, it briefly explains the phenomenon that the authors would like to investigate. However, it fails to provide any more details on the mentioned models of mental sampling. The authors go through the models and compare them in two paragraphs, which, in my opinion, is not enough for a person who does not know much about them. They should have at least a section in which they describe them and compare them. The same should be done with the random series generation. The authors mention a couple of papers on the topic, while there is a rich literature on the topic. I put a few papers below that the authors could find relevant to their work.

Finally, the authors fail to highlight the hypothesis or even a research question they would like to investigate. At the end of the introduction, they just state the following: "The current paper thus uses random generation to access the sampling process underlying decision making. We report two experiments applying random generation to the common decision-making paradigm of risky choice: participants were presented with pairs of monetary gambles and repeatedly produced their possible outcomes before choosing between them. These generated sequences were then examined for key patterns suggested by the proposed decision-making models, as well as their relation to subsequent choices." I understand that it is more of an exploratory paper than testing a specific hypothesis, but I assume that, based on the models mentioned in the introduction, they could at least try to verify which one is the closest to what people are actually doing. I would expect authors to state more clearly what they expect to see in the results. So far, in the results section, they perform some analysis without a clear explanation of why they do them.

However, my main concern is that the authors keep referring to random generation, while as far as I understand the

description of the task, participants in only one pair were asked to produce a random series. In most cases, they were to produce a series from a non-uniform distribution; therefore, the fact that the results deviate from a random one is hardly surprising. For example, when computing the autocorrelation, I am not sure if I understand why the authors compare the empirical results against a random shuffle and not the results drawn from a given distribution of the gamble score. The results of their comparison show how far the given result is from a random shuffle, while in my opinion, it would be worth checking whether people were able to recreate the given distribution in terms of the autocorrelation.

In general, the presentation of the results is quite unclear. The authors keep coming up with dependent variables with no good definition of how they are defined. It would really help if they provided either an analytical strategy section or at least described how the dependent variables were computed and what they show. The same could be said about the independent variables.

In my opinion, the paper requires rewriting. For me, after reading it carefully a couple of times, it is still not clear what exactly the authors wanted to investigate and why they performed the analysis they did. Below, please find some more detailed comments on specific parts.

Detailed comments:
The explanation of the sample size, "We aimed for a sample size of 50 participants to roughly correspond with counts used in previous random generation studies (e.g., [21,22])" is less than convincing. Especially considering that both of the cited papers, among which one is a preprint, are written by the authors. Power analysis would be welcomed here.

To my understanding, people were generating values based on the gamble score they were shown. They had a maximum of 1 minute to generate one value and 2 minutes per pair? I am not sure why participants had to type the value instead of choosing between the given values, for example, either type 1 or 101? What was the purpose behind it? And pressing simultaneously "Z" and "M" keys to confirm each guess? Please see Annand & Holden (2022), as they provide a possible justification for this procedure. However, this might somehow limit a natural tendency for the response.

It would really help if the authors explained the effects presented in Table 2. What exactly does the results column say? What is the expected result? I understand it is based on the research by Erev et al. (2017), but at least a brief description of their results would be welcomed. So far, the author's manuscript feels a bit like an addition to the work of Erev et al. (2017). Even a brief description of their main results would be beneficial for a potential reader.

The authors only report the results of Linear regression in text with no table summarizing the results. Therefore, it is, for example, difficult to understand whether the intercept was significant or not. Similarly, what do the authors mean by "Subsequent comparisons did however find this slope was significantly below 1 in both cases (E1: $t(1215) = -4.37$, $p < .001$; E2: $t(1085) = -6.18$, $p < .001$), implying a general bias in generation rates: low probability events were over-generated and high probability events were under-generated."? (lines 131-133). Do they mean the intercept?

I have hard time in understanding how the authors report the results, for example in lines from 155 to 160 they state the following "This found no significant difference from a slope of 1 in either task (E1: B = 1.10 [0.93 1.27], $t(21) = 1.18$, $p = .251$; E2: B = 0.93 [0.72, 1.13], t(21 = -.77, $p = .451$), providing no ovidence of the bias shown in overall generation rate. This difference is further supported by comparing the confidence intervals of these slopes against the main effects across all responses reported above: for both experiments, each coefficient is well outside the other's interval, suggesting a distinct effect of probability in the first response." To my understanding, first they say they did not find the difference from a slope of 1 and afterward state that this "(...) difference is further supported (...)". Which is it then?

Literature
Oskarsson, A. T., Van Boven, L., McClelland, G. H., & Hastie, R. (2009). What's next? Judging sequences of binary events. Psychological Bulletin, 135(2), 262–285. https://doi.org/10.1037/a0014821
Williams, J. J., & Griffiths, T. L. (2013). Why are people bad at detecting randomness? A statistical argument. Journal of Experimental Psychology: Learning, Memory, and Cognition, 39(5), 1473–1490. https://doi.org/10.1037/a0032397
Nickerson, R. S. (2002). The production and perception of randomness. Psychological Review, 109(2), 330–357. https://doi.org/10.1037/0033-295X.109.2.330
Schulz, M.-A., Schmalbach, B., Brugger, P., & Witt, K. (2012). Analysing Humanly Generated Random Number Sequences: A Pattern-Based Approach. PLOS ONE, 7(7), e41531. https://doi.org/10.1371/journal.pone.0041531
Annand, C. T., & Holden, J. G. (2022). Embodied nonlinear dynamics of cognitive performance. Journal of Experimental Psychology: General. https://doi.org/10.1037/xge0001319
Biesaga, M., Talaga, S., & Nowak, A. (2021). The Effect of Context and Individual Differences in Human-Generated Randomness. Cognitive Science, 45(12), e13072. https://doi.org/10.1111/cogs.13072
Biesaga, M., & Nowak, A. (2024). The role of the working memory storage component in a random-like series generation. PLOS ONE, 19(1), e0296731. https://doi.org/10.1371/journal.pone.0296731
Vandierendonck, A., Demanet, J., Liefooghe, B., & Verbruggen, F. (2012). A chain-retrieval model for voluntary task switching. Cognitive Psychology, 65(2), 241–283. https://doi.org/10.1016/j.cogpsych.2012.04.003

Reviewer #2 (Remarks to the Author):

This paper studies the kind of mental sampling process that has become increasingly influential in various models of decision making. The authors develop an experimental paradigm with choice between risky gambles in which the (typically

latent) samples representing the possible outcomes are explicitly elicited. Across two online experiments (total N = 103), participants repeatedly generated potential outcomes from pairs of monetary gambles before choosing between them. The authors used these “random generation” sequences to infer how internal sampling might deviate from objective probabilities, serial independence, or have biased started points. In relation to their generated sequences, they find that participants over-generated rare outcomes, under-generated common ones and avoided repeating values. In relation to choice, they find no significant difference for nearly all decision algorithms in predicting actual participant choice when using the generated sequences than when using the described objective probabilities, except for the ‘higher mean’ algorithm, which was significantly greater when using the participant generated sequences.

The studies are novel and endeavor to bear on a general class of models, which speaks in their favor. The writing is clear, and the analyses are generally well performed. I do have some issues with the interpretation of results, which substantially limits their meaning and applicability, and should be recognized before the paper is ready for publication.

The fundamental assumption upon which the most interesting claims of this paper rest is that participant reported sequences actually mirror the mental sampling that might be taking place. However, it is not clear that this is the case here. Presumably, mental sampling particularly of simple distributions is much faster than participants are able to report in this experiment, and there is much more that goes into participants finally reporting the samples. Perhaps the reported samples are more about communication – participants are trying to communicate the distribution to another person via the samples? Perhaps is it about trying to emulate randomization – participants are trying to generate samples that seem to be random for the specified distribution? Both of these processes are quite separate from “mental sampling”. If there were a stronger correspondence between mental sampling and the reported sequences, I think we would see stronger results predicting actual choice. This is a major limitation of the study and the authors should discuss it in much more depth. Assuming that these reports aren’t at all related to actual mental sampling in the decision process, how would we interpret the results of the study? What is its worth? Assuming it is noisy, but bears some resemblance to actual mental sampling, how might we interpret the results then?

Many sampling-based theories of the decision making process are meant to represent processes where memory plays a big role, most notably query theory. However the task here uses numerical gambles, and presents the gambles throughout, therefore limiting the scope for memory biases. This doesn’t completely ruin the premise since some models like DFT and other DDMs can be based on stimuli that are always present, but it may help explain why few distortions are found and why the samples have low added predictive power.

Because of the setup, I suspect that a number of the observed findings are due to a different unmentioned mechanism that has to do with communicating the distribution. Take the case where one outcome has a 1% probability of occurring. Given that people generate about 25 samples, the closest option to reflect the true distribution would be to report 0 samples. This feels obviously lacking when one is trying to convey the distribution to others. A participant may then sensibly throw in a single sample to demonstrate that it exists, even though this will look like overestimation of low probability events. So it may not indicate an error in probability judgment but a property of communicating representative samples. This notion would be consistent with the tendency to start generating high probability events for example. It would drastically alter the interpretation of results, and think this must be mentioned centrally.

A few papers/literatures are perhaps relevant but not cited. Ronayne and Brown (2017, Journal of Mathematical Psychology) explicitly elicit the distributions of items in multi-attribute choices based on the choice set. There is also a sizeable literature in experimental economics on generation of random sequences (pertaining to mixed strategies in game theory) reviewed in Camerer (2003). This could also help explain some of the underrepetition if people are trying to convey randomness.

Other comments:

Was the number of about 25 samples generated for 2 gambles or each one in a trial? More explicit details on the elicitation process would make things clearer, even though they are not complicated.

If the responses for singular outcomes should always be the same, did everybody left after exclusions indeed follow this?

In Figure 2 what do the conditional medians look like?

It would help readers to explain the autocorrelation function calculations in more detail, maybe with an example.

Figure A1 is interesting and should at the least be mentioned more prominently in the main text.

How well do the samples predict choice for the pre-sample choice? Maybe I missed it, but it isn’t clear to me whether, for experiment 2 it is the pre or post score that is reported in the paper. I’m assuming it’s the post to be the same as experiment 1 – but regardless it’d be good to see both.

The authors should consider discussing the methods before the results.

Reviewer #3 (Remarks to the Author):

I co-reviewed this manuscript with one of the reviewers who provided the listed reports. This is part of the Communications Psychology initiative to facilitate training in peer review and to provide appropriate recognition for Early Career Researchers

who co-review manuscripts.

Reviewer #4 (Remarks to the Author):

Review
Mental Sampling in Preferential Choice: Tracing the Process using Random Generation

While many decision making models assume some form of mental sampling process, the specifics of this sampling process often remain debated. The article suggests accessing the internal sampling process through the explicit generation of outcomes. The authors present a series of analyses on how people generate outcomes from described risky gambles and how these generated outcomes relate to subsequent choices. Participants generated more low-probability outcomes and fewer high-probability outcomes than warranted by objective probabilities. Results on alternation align with previous work on random generation of binary sequences, showing that people's generated outcome sequences tend to deviate from truly random processes. Yet, the predictive power of generated outcomes on choice—instead of true outcome probabilities—seems to be minimal.

The article is well-written, the analyses seem appropriate and the idea to link mental sampling processes with random generation of outcomes—in particular, from described risky gambles—seems to be a novel contribution to the literature. That being said, there are some major concerns about the underlying hypotheses, theoretical assumptions, and study design that may limit the contribution of the article to the field.

1. The research question is rather vague and leaves some room for interpretation about the goal of the study ("The current paper thus uses random generation to access the sampling process underlying decision making", p. 5, line 89). Similarly, there are no clearly stated hypotheses. If this study is, indeed, fully exploratory in nature, it might make sense to state this clearly and possibly collect more data to substantiate the evidence given specific hypotheses.
2. The authors' main conclusion seems to be that the presented "findings suggest systematic biases in mental sampling" (p. 2, line 25-26). As such, the study builds on the assumption that random generation can appropriately trace mental sampling processes. However, the article is missing explanations and/or the discussion of (previous) evidence that convincingly establishes this relationship. Moreover, the authors state the possibility that "explicit generations [...] do not reflect the implicit evidence considered by decision makers [...] (p. 18, line 366-378) and continue to say that this is even implied by their results. Together, this seems to be a bit confusing: (1) can the authors convincingly establish the assumed relationship between mental sampling and random generation, or (2) did they a priori expect discrepancies between random generation and mental sampling, or (3) is this even the research question they set out to explore? The article may serve as an interesting contribution to clarifying the relationship between random generation and mental sampling, but this may require considerable reframing of the introduction.
3. The authors highlight results from Experiment 2 as a novel contribution, suggesting that asking people to randomly generate outcomes alters their choices (from a pre- to a post-test). To substantiate this claim and justify any conclusions that follow from it, the analyses require an active/passive control condition.
4. Information on the mechanistic aspects of how people generate outcomes from described probabilities is largely omitted in the article. However, this could be an interesting aspect that may be worthwhile to elaborate on and that could more explicitly inform the random generation–mental sampling relationship, as well as the references to different computational cognitive models. What cognitive processes do the authors suggest are at play?

Minor
- The 2-minute time limit for random generation and restriction to generate outcomes in an alternating fashion make sense from a practical perspective, but could have considerable effects on the results. Given the work on optional stopping in sampling paradigms, it could be interesting to compare this condition with one that allows people to generate as many outcomes as they think best represent the gamble and to allow for non-alternating outcome generations.
- Described risky gambles are arguably not extremely common in people's everyday lives. Without experience with what random outcome sequences of these risky gambles may look like, people could draw on their own (biased) experiences of random processes, which may hold some properties that are different from risky gambles (e.g., autocorrelation, sequential dependencies) and some that may be similar to the risky gambles used in this study (e.g., negative risk-reward relationship). Literature on the description–experience gap and more plausible statistical structures of real-world environments might be helpful starting points to put some of the presented findings in context beyond risky gambles with described probabilities.
- We would like to encourage the authors to be a bit more specific in explaining the performed analyses, in particular, the random effects structure of the regression models (i.e., participant-specific slopes and intercepts) and how the (in-)dependent variables in these models came about (particularly if not explained in the CSV_guide.txt). For instance, it seems unintuitive that "start rate" is not an actual rate but a newly created binary variable (the label is particularly confusing in Figure 2), which does not seem to be explained in the manuscript. A dedicated section in the "Methods" part could maintain the brevity of the results section if needed.
- The caption in Table 1 could benefit from some more explanation, for instance, that "B choice rate" refers to the risky option, whereas "A" is the safe option in most cases, except for Gamble 2 where both options are risky.
- Table 2: Please describe the numbers under "A" and "B" as outcome and probability in the caption.
- Please indicate the age unit (here: year). Given the large age range and previous work pointing to age differences in preferential choice, for instance, in decision quality and risk aversion, it might be informative to include age as a covariate. Otherwise, this may be an issue worth discussing.
- Please spell out "CI" in the caption of Figure 4 to avoid confusion with credible intervals.

- Did you perform any attention checks and any measures to prevent bots/ people using bots from participating in your study?
- Please add the unit of the reaction times to the data documentation. Looking at the reaction times, there seem to be some additional participants who might not have paid much attention to the task (many trials with generation reaction times of what seems to be well under 1 second, with streaks of alternating or the same outcomes).

Reviewer #5 (Remarks to the Author):

I co-reviewed this manuscript with one of the reviewers who provided the listed reports. This is part of the Communications Psychology initiative to facilitate training in peer review and to provide appropriate recognition for Early Career Researchers who co-review manuscripts.

* TRANSPARENT PEER REVIEW: Communications Psychology uses a transparent peer review system. This means that we publish the editorial decision letters including Reviewers' comments to the authors and the author rebuttal letters online as a supplementary peer review file. However, on author request, confidential information and data can be removed from the published reviewer reports and rebuttal letters prior to publication. If your manuscript has been previously reviewed at another journal, those Reviewers' comments would not form part of the published peer review file.

** Visit Nature Research's author and referees' website at www.nature.com/authors for information about policies, services and author benefits**

Communications Psychology is committed to improving transparency in authorship. As part of our efforts in this direction, we are now requesting that all authors identified as ‘corresponding author’ create and link their Open Researcher and Contributor Identifier (ORCID) with their account on the Manuscript Tracking System prior to acceptance. ORCID helps the scientific community achieve unambiguous attribution of all scholarly contributions. You can create and link your ORCID from the home page of the Manuscript Tracking System by clicking on ‘Modify my Springer Nature account’ and following the instructions in the link below. Please also inform all co-authors that they can add their ORCIDs to their accounts and that they must do so prior to acceptance.
https://www.springernature.com/gp/researchers/orcid/orcid-for-nature-research

For more information please visit http://www.springernature.com/orcid

If you experience problems in linking your ORCID, please contact the Platform Support Helpdesk.

Version 1:

Decision Letter:

** Please ensure you delete the link to your author homepage in this e-mail if you wish to forward it to your coauthors **

Dear Dr Spicer,

Your manuscript titled "Generated Outcomes in Risky Choice Reveal Biased Sampling and Sequential Dependencies" has now been seen by our reviewers, whose comments appear below.

Reviewer #4 has significant concerns regarding the evidentiary strength of the manuscript in its current form. We discussed these concerns with one of the other members of the panel of referees, who expressed the view that while the concerns were warranted, and that there are limitations the generalizability of the results, the insights that the work offers do make a significant contribution to the field. In revision, we require a more caveated presentation of the inferences supported by the results, but you are not required to add more empirical work.

In light of their combined advice we are happy, in principle, to publish a suitably revised version in Communications Psychology.

We therefore invite you to revise your paper one last time to address the remaining concerns of our reviewers, especially regarding the limitations of the approach used, and a list of editorial requests. At the same time we ask that you edit your manuscript to comply with our format requirements and to maximise the accessibility and therefore the impact of your work.

EDITORIAL REQUESTS:

Please review our specific editorial comments and requests regarding your manuscript in the attached "Editorial Requests Table". Please outline your response to each request in the right hand column. Please upload the completed table with your manuscript files as a Related Manuscript file.

If you have any questions or concerns about any of our requests, please do not hesitate to contact me.

SUBMISSION INFORMATION:

In order to accept your paper, we require the files listed here https://www.nature.com/documents/commsj-file-checklist.pdf .

OPEN ACCESS:

Communications Psychology is a fully open access journal. Articles are made freely accessible on publication. For further information about article processing charges, open access funding, and advice and support from Nature Research, please visit https://www.nature.com/commspsychol/open-access

At acceptance, you will be provided with instructions for completing the open access licence agreement on behalf of all authors. This grants us the necessary permissions to publish your paper. Additionally, you will be asked to declare that all required third party permissions have been obtained, and to provide billing information in order to pay the article-processing charge (APC).

* TRANSPARENT PEER REVIEW: Communications Psychology uses a transparent peer review system. On author request, confidential information and data can be removed from the published reviewer reports and rebuttal letters prior to publication. If you are concerned about the release of confidential data, please let us know specifically what information you would like to have removed. Please note that we cannot incorporate redactions for any other reasons.

* CODE AVAILABILITY: All Communications Psychology manuscripts must include a section titled "Code Availability" at the end of the methods section. We require that the custom analysis code supporting your conclusions is made available in a publicly accessible repository at this stage; please choose a repository that generates a digital object identifier (DOI) for the code; the link to the repository and the DOI must be included in the Code Availability statement. Publication as Supplementary Information will not suffice.

* DATA AVAILABILITY:
All Communications Psychology manuscripts must include a section titled "Data Availability" at the end of the Methods section. More information on this policy, is available in the Editorial Requests Table and at http://www.nature.com/authors/policies/data/data-availability-statements-data-citations.pdf.

Please use the following link to submit the above items:
Link Redacted
** This url links to your confidential home page and associated information about manuscripts you may have submitted or be reviewing for us. If you wish to forward this email to co-authors, please delete the link to your homepage first **

We hope to hear from you within two weeks; please let us know if you need more time.

Best regards,

Jennifer Bellingtier

Jennifer Bellingtier, PhD
Senior Editor
Communications Psychology

Caroline Charpentier, PhD
Editorial Board Member
Communications Psychology
orcid.org/0000-0002-7283-0738

REVIEWER EXPERTISE:
Reviewer #1: mental sampling, random generation processes

Reviewers #2 and #3 (co-review with ECR): mental sampling, risky decision-making
Reviewers #4 and #5 (co-review with ECR): risky decision-making

REVIEWERS' COMMENTS:

Reviewer #1 (Remarks to the Author):

I am happy with the reviews done by the authors. All my concerns and doubts were addressed. Having said that, I would suggest that authors consider splitting the introduction into shorter sections. This is not a necessity since, as it is now, this reads well, but rather something to consider.

While I think the manuscript is suitable for publication, I would like the authors to address in the final version one detail that raised my concern when reading the reviewed manuscript. That is the description of the gratification for the participants. To my understanding, there was a fixed rate for the participation and a bonus within a range between £0 and £15 (the range is only explicitly stated in Study 1, but I assume in Study 2 it was the same). However, I am not sure how exactly the upper bound of the bonus was calculated. As far as I understand, it should be 5% of the highest possible win. According to Table 1, the highest possible win is £256, and consequently, the highest possible bonus is £12.8, not £15.

Moreover, the actual average of the bonus in Study 1 was £0.33 (so the sum of all bonuses was around £17), and in Study 2, it was £0.41 (so it adds up again to around £17). While I understand that money in science does not grow on trees, I would suggest in future studies to be more explicit about what the participants can expect because. In my opinion, it is a bit misleading to say that the bonus could be up to £15 while the actual chances of being even close to such an amount are negligible. Moreover, the total sum of the bonuses for all participants was only slightly bigger than the upper bound of the declared possible bonus...

Reviewer #2 (Remarks to the Author):

Thanks to the authors for a thoughtful revision. The difference between mental and reported samples has now been made more explicit in the paper. My comments are almost entirely addressed.

p. 31: “participants may have sought to use their generations to communicate the gambles’ distribution of outcomes to a third party” It need not be a third party, if the experimenters themselves are the second party.

Regarding Figure 2, I did indeed mean the “median generation rate within each true probability rather than the means”.

Reviewer #3 (Remarks to the Author):

I co-reviewed this manuscript with one of the reviewers who provided the listed reports. This is part of the Communications Psychology initiative to facilitate training in peer review and to provide appropriate recognition for Early Career Researchers who co-review manuscripts.

Reviewer #4 (Remarks to the Author):

Mental Sampling in Preferential Choice: Tracing the Process using Random Generation

Review after Revision 1
We appreciate the effort invested in revising the manuscript and note clear improvements in several sections (e.g., the introduction on random generation, rule analyses, and the discussion). However, the revision remains largely superficial with respect to the central issues flagged previously. Two core concerns persist and make it difficult to establish strong claims: (a) conceptual clarity about the link between mental sampling and random generation, and (b) the adequacy and credibility of the evidence—especially the pre-/post-generation choice difference in Experiment 2.

The manuscript still treats random generation and mental sampling as more tightly linked than the evidence may warrant; the response letter and revisions do not yet resolve this ambiguity. While it seems to be an interesting avenue to investigate whether explicit generation reflects mental sampling, parts of the manuscript read as if this link were already established (e.g., line 118: “The advantage of random generation in choice settings is that this procedure mirrors the mental sampling assumed in the decision making theories noted above”). Elsewhere, “random/explicit generation” appears to already be replaced by “mental sampling” (e.g., abstract, line 20: “These findings suggest systematic biases in mental sampling...” when referring to the results from the random generation task). Further conceptual inconsistencies arise from the findings of Experiment 2 and the discussion thereof. If random generation both mirrors mental sampling and causally alters subsequent choice, how should we understand the process underlying “pre-generation” choice? Does pre-generation choice entail no mental sampling, or is a qualitatively different sampling process involved, or does this pattern suggest that random generation is not an appropriate proxy for mental sampling more generally speaking? This would be important to clarify in light of the implications for the computational models the authors seek to evaluate.

The re-analysis of secondary data (i.e., Erev et al., 2017) is a useful addition to the pre-/post-generation choice difference

and may serve as a starting point to generate testable hypotheses. However, it is not sufficient to substantiate the claims implied by the observed pre-/post-generation choice difference, given substantial methodological and sample differences. The manuscript itself acknowledges that the present data may not yet yield the desired credibility (discussion, line 562: “further contrasts […] will be needed to confirm whether this is a reliable result”). We strongly encourage the authors to run separate control/experimental conditions that may provide more informative insights into the underlying process, for instance, whether the time between choices or extended exploration of the outcome space may provide more explanatory power for altering choice. When collecting additional data, it seems advisable to integrate standard measures for attention and bot checks.

Other comments
Exploratory status: The newly added research questions suggest there were no a priori formalized predictions. If so, please state clearly that the work is exploratory, so readers can appropriately weigh the evidence and the role of post hoc interpretation.
Likelihood ratios vs Bayes factors: As noted (lines 484–485), the reported quantities are likelihood ratios, not Bayes factors (which require marginal likelihoods over priors). To avoid confusion, please label them “likelihood ratios” and, if desired, note their interpretive analogy to inclusion Bayes factors.

In sum, the manuscript has improved in presentation, but core conceptual and evidential issues remain unresolved. Substantial revision—particularly clarifying the construct mapping and providing new data—is necessary before strong conclusions can be drawn.

Reviewer #5 (Remarks to the Author):

I co-reviewed this manuscript with one of the reviewers who provided the listed reports. This is part of the Communications Psychology initiative to facilitate training in peer review and to provide appropriate recognition for Early Career Researchers who co-review manuscripts.

** Visit Nature Research's author and referees' website at www.nature.com/authors for information about policies, services and author benefits**

Open Access This Peer Review File is licensed under a Creative Commons Attribution 4.0 International License, which permits use, sharing, adaptation, distribution and reproduction in any medium or format, as long as you give appropriate credit to the original author(s) and the source, provide a link to the Creative Commons license, and indicate if changes were made.
In cases where reviewers are anonymous, credit should be given to 'Anonymous Referee' and the source.
The images or other third party material in this Peer Review File are included in the article’s Creative Commons license,

unless indicated otherwise in a credit line to the material. If material is not included in the article's Creative Commons license and your intended use is not permitted by statutory regulation or exceeds the permitted use, you will need to obtain permission directly from the copyright holder.
To view a copy of this license, visit https://creativecommons.org/licenses/by/4.0/

COMMSPSYCHOL-25-0744-T Reviewer Responses

Editor

Dear Dr Spicer,

Thank you for your patience during the peer-review process. Your manuscript titled "Mental Sampling in Preferential Choice: Tracing the Process using Random Generation" has now been seen by 5 reviewers, whose comments are appended below. You will see that they find your work of some potential interest. However, they have raised quite substantial concerns that must be addressed.

In light of these comments, we cannot accept the manuscript for publication but would be interested in considering a revised version that fully addresses these serious concerns. Please bear in mind that we will be reluctant to approach the reviewers again in the absence of substantial revisions.

If you choose to take up this option, please highlight all changes in the manuscript text file, and provide a detailed point-by-point reply to the reviewers.

Editorially, we consider it important that the revised manuscript addresses the conceptual and methodological concerns of the reviewers through additional description, analysis, and experimentation where needed. Please clarify the rationale for the research question and hypotheses, strengthen or discuss the theoretical assumptions brought up by the reviewers (related to random generation, to mental sampling, and their association), and provide more methodological clarify.

Thank you for providing us with an opportunity to revise our paper. We have substantially revised the manuscript to answer the reviewers' comments, adding more explicit research questions, expanding on both existing mental sampling models and past uses of random generation, and clarifying our assumptions on the link between generation and mental sampling. We have also amended our Methods and Results to better convey our experimental design and analyses, and added additional discussion of the limitations of our study. Our revised manuscript also includes a more sensitive test of the link between generations and choices to strengthen the case for a connection between these responses. We hope these changes answer our reviewers' concerns.

Please ensure you follow our statistical guidelines when reporting statistics (https://www.nature.com/commspsychol/submit/submission-guidelines#statistical-guidelines). Please note in particular our requirements for the reporting and interpretation of null-results. Non-significant findings derived from null-hypotheses significance tests should be reported in full, but may not be interpreted. Where you interpret null results, this interpretation must be based on Bayes Factors or equivalence tests.

We have endeavoured to follow these guidelines to the best of our ability, but are happy to make further amendments if necessary.

If you decide to revise the manuscript in response to these issues, please also implement all requests in the attached Mandatory Revision Requests document. All requirements listed in this document need to be fully met, or the work will be returned to you for further revisions without peer review. This workflow is in place to increase the likelihood that the paper will ultimately be accepted for publication. It reduces the number of rounds of revision (and review) and ensures that the reviewers vet a version of the article that is compliant with

journal policies. If you have any questions regarding the required revisions, please contact the journal prior to resubmission to avoid a negative outcome.

We have added all the requested changes from the attached form, and hope the paper now fully follows the journal's standards.

If the revision process takes significantly longer than five months, we will be happy to reconsider your paper at a later date, provided it still presents a significant contribution to the literature at that stage.

We would appreciate it if you could keep us informed about an estimated timeline for resubmission, to facilitate our planning.

Please submit the following items:

- Revised manuscript

- Point-by-point response to the referees' comments

- Mandatory Revision Requests Table (attached).

- Cover letter (as a separate document)

Thank you for the opportunity to review your work.

Best regards,

Caroline Charpentier

REVIEWER EXPERTISE:

Reviewer #1 mental sampling, random generation processes

Reviewer #2 mental sampling, risky decision-making

Reviewer #4 risky decision-making

REVIEWER REPORTS

Reviewer #1

In the manuscript, the authors investigate the sampling process in the mental sampling mechanism. In two studies, they asked participants to generate elements from 8 different distributions and compare their results against series generated at random. Their results

showed over-generation of rare elements while under-generation of common outcomes. Moreover, they showed the avoidance of the direct repetition of elements. Their finding suggest that the process of mental sampling is not driven by a random process.

In general, the paper is difficult to follow. While the authors try to follow Springer's structure of the paper by having the detailed description of the methods after the results, it makes the paper hard to comprehend. For example, the brief introduction of the procedure before the results, in my opinion, is not enough to understand exactly the task participants had to follow. I would suggest having a more traditional presentation of the method section with a detailed description of the procedure before the results (as far as I know, it is not mandatory to follow this one).

We apologise for any confusion caused by the structure of the manuscript: indeed, we sought to follow Springer's format, but understand this can interfere with comprehension. As suggested, we have reordered the paper to place the Methods ahead of the Results, which we believe does improve the clarity of the paper.

When it comes to the structure of the paper, I would suggest expanding the introduction. So far, it briefly explains the phenomenon that the authors would like to investigate. However, it fails to provide any more details on the mentioned models of mental sampling. The authors go through the models and compare them in two paragraphs, which, in my opinion, is not enough for a person who does not know much about them. They should have at least a section in which they describe them and compare them. The same should be done with the random series generation. The authors mention a couple of papers on the topic, while there is a rich literature on the topic. I put a few papers below that the authors could find relevant to their work.

Thank you for this suggestion, we understand our summary of these models was somewhat brief for unfamiliar readers. We have expanded our Introduction to offer more detail on existing mental sampling models; these are higher level summaries rather than full technical descriptions, but we hope these highlight the key differences in their operation which inform the aims of our study.

We have also added more detail on past uses of random generation, including some of the suggested references, for which we are grateful. We agree that there is a substantial literature on this subject, though this prior work does have different goals to our study: past uses of random generation commonly focus on people's ability to emulate true randomness, whereas we use this task as a tool to access the sampling algorithm underlying choice. While there are some common elements between these aims (such as evaluating independence in generations), we now better delineate our use of generation tasks from this literature, and the motivations behind our analyses.

Finally, the authors fail to highlight the hypothesis or even a research question they would like to investigate. At the end of the introduction, they just state the following: "The current paper thus uses random generation to access the sampling process underlying decision making. We report two experiments applying random generation to the common decision-making paradigm of risky choice: participants were presented with pairs of monetary gambles and repeatedly produced their possible outcomes before choosing between them. These generated sequences were then examined for key patterns suggested by the proposed decision-making models, as well as their relation to subsequent choices." I understand that it is more of an exploratory paper than testing a specific hypothesis, but I assume that, based on the models mentioned in the introduction, they could at least try to verify which one is the closest to what people are actually doing. I would expect authors to state more clearly what they expect to see in the results. So far, in the results section, they perform some analysis without a clear explanation of why they do them.

Thank you, this is an important point. We did not provide specific hypotheses in our previous submission as any expectations are model-dependent, and we did not wish to commit to one particular model: our study aimed to investigate the assumptions of these models whilst remaining impartial, identifying key properties across models in the Introduction which could be examined in our generation tasks. We do appreciate however that this text was not sufficiently explicit to fully motivate our analyses, and have now added specific research questions to the end of the Introduction detailing the features we intended to test in our experiments. While these are not labelled as 'hypotheses' as they are not explicit predictions, we hope this better conveys the intentions of our study. We also note the correspondence between our results and existing sampling models in the Discussion, though we avoid declaring a single 'winning' model as our study indicates the features of the underlying sampling algorithm but does not definitively identify it.

However, my main concern is that the authors keep referring to random generation, while as far as I understand the description of the task, participants in only one pair were asked to produce a random series. In most cases, they were to produce a series from a non-uniform distribution; therefore, the fact that the results deviate from a random one is hardly surprising. For example, when computing the autocorrelation, I am not sure if I understand why the authors compare the empirical results against a random shuffle and not the results drawn from a given distribution of the gamble score. The results of their comparison show how far the given result is from a random shuffle, while in my opinion, it would be worth checking whether people were able to recreate the given distribution in terms of the autocorrelation.

Thank you for this comment, this is indeed an important point to clarify. We apologise for any confusion caused by our use of the term 'random generation': we took this label from past studies to refer to tasks in which participants stochastically produce values from a given distribution, aiming to provide a simple and accessible name for this procedure suitable for a general audience. As you note, these past studies focused on uniform distributions following a definition of 'random' based on equiprobable outcomes, but such a definition is not central to our study: we use these tasks to access mental sampling processes, which can apply to varying distributions. Our use of this term thus sought to reflect the similarity of our *procedure* to previous random generation tasks, if not the specific *stimuli*. This also coincides with how this term is commonly used in computer science and statistics. This being said, we appreciate that this can cause confusion for readers more familiar with previous uses of random generation, and so have edited the Introduction to clarify this distinction, and reduced our use of the 'random' label throughout the paper.

Following on from this, our comparisons are not against the standard of a uniform distribution, but rather the true distribution given in the gamble description. Regarding the autocorrelation analyses, the shuffled series are used to control for any deviation of the empirical distribution from the objective distribution to provide a more direct baseline for independence. The true autocorrelation of these distributions is also somewhat ambiguous as each gamble is only ever going to be played once: our contrast is against independence as that is more commonly assumed by mental sampling models. We have edited the description of this procedure to clarify this reasoning.

In general, the presentation of the results is quite unclear. The authors keep coming up with dependent variables with no good definition of how they are defined. It would really help if they provided either an analytical strategy section or at least described how the dependent variables were computed and what they show. The same could be said about the independent variables.

We apologise for any confusion in our results: while the primary data collected in our experiments are the sequences generated by the participants, our analyses take multiple

measures from these series to examine the statistical signatures predicted by existing mental sampling models. We have added a statement on this to the Design and Materials section of our Methods, and edited the Results to more concretely introduce each dependent variable. We also hope our explicit research questions help set out the goals of our analyses earlier in the paper.

In my opinion, the paper requires rewriting. For me, after reading it carefully a couple of times, it is still not clear what exactly the authors wanted to investigate and why they performed the analysis they did. Below, please find some more detailed comments on specific parts.

We apologise for any lack of clarity in the previous submission, and hope our addition of explicit research questions and further detail on our methods and analyses helps solve these issues.

Detailed comments:

- The explanation of the sample size, "We aimed for a sample size of 50 participants to roughly correspond with counts used in previous random generation studies (e.g., [21,22])" is less than convincing. Especially considering that both of the cited papers, among which one is a preprint, are written by the authors. Power analysis would be welcomed here.

This is a good point: because our study uses a fairly novel experimental paradigm, a priori power analyses are difficult, particularly given our use of mixed model regressions which do not have simple analytical solutions for statistical power. We have however added post-hoc power analyses to the paper showing that our sample size was sufficient to detect medium effect sizes with a minimum power of 0.934 across our analyses.

- To my understanding, people were generating values based on the gamble score they were shown. They had a maximum of 1 minute to generate one value and 2 minutes per pair? I am not sure why participants had to type the value instead of choosing between the given values, for example, either type 1 or 101? What was the purpose behind it? And pressing simultaneously "Z" and "M" keys to confirm each guess? Please see Annand & Holden (2022), as they provide a possible justification for this procedure. However, this might somehow limit a natural tendency for the response.

We apologise for any confusion regarding our task design: to clarify, each trial presented participants with a pair of gambles, and participants generated multiple possible payouts from both gambles, alternating between the two options, for two minutes per pair. The 1 minute likely refers to the 60 second limit applied to each response (fitting the total two minutes per pair) to ensure the task progressed automatically. We used typed outcomes to ensure participants were aware of the value of the payouts in each gamble, which risk being overlooked if using index-like responses, and now note this in the manuscript. The “Z” and “M” combination for confirmation was used to force participants to move their fingers between responses to discourage them from simply hammering the same key, which we have also clarified in the Methods.

- It would really help if the authors explained the effects presented in Table 2. What exactly does the results column say? What is the expected result? I understand it is based on the research by Erev et al. (2017), but at least a brief description of their results would be welcomed. So far, the author's manuscript feels a bit like an addition to the work of Erev et al. (2017). Even a brief description of their main results would be beneficial for a potential reader.

We apologise for any confusion with this table: we have relabelled the ‘Result’ column as ‘Expected Preference’ to better convey that this is the choice pattern reflecting each effect, and added a new column listing the choice rate from Erev et al. (2017) as an illustration (please note that this is now Table 1 due to the reordering of the Methods section). We have also added a new Appendix A offering more detailed descriptions of each effect.

- The authors only report the results of Linear regression in text with no table summarizing the results. Therefore, it is, for example, difficult to understand whether the intercept was significant or not. Similarly, what do the authors mean by "Subsequent comparisons did however find this slope was significantly below 1 in both cases (E1: $t(1215) = -4.37$, $p < .001$; E2: $t(1085) = -6.18$, $p < .001$), implying a general bias in generation rates: low probability events were over-generated and high probability events were under-generated."? (lines 131-133). Do they mean the intercept?

Thank you for this suggestion: we appreciate that specific results can be hard to pull from the text, and so have collected the regression statistics into a new Table 2. We did not examine the intercept in our previous submission as our focus is on sensitivity to the objective probabilities given in the gamble descriptions, which is primarily captured by the slope in these regressions, though we do now include intercepts in the new table. Additionally, because these analyses examine the rates of mutually exclusive events which must sum to one, slopes below one necessitate that intercepts are above zero. The quoted text refers to our use of multiple comparisons of this estimated slope, which may be the source of the confusion: first, the slope is compared against a null of 0 to test for *any* sensitivity (which is rejected in both experiments); next, we compare the slope against a null of 1 reflecting perfect following of objective information (which is also rejected). We have amended the text here to better clarify this process.

- I have hard time in understanding how the authors report the results, for example in lines from 155 to 160 they state the following "This found no significant difference from a slope of 1 in either task (E1: $B = 1.10$ [0.93 1.27], $t(21) = 1.18$, $p = .251$; E2: $B = 0.93$ [0.72, 1.13], $t(21 = -.77$, $p = .451$), providing no ovidence of the bias shown in overall generation rate. This difference is further supported by comparing the confidence intervals of these slopes against the main effects across all responses reported above: for both experiments, each coefficient is well outside the other's interval, suggesting a distinct effect of probability in the first response." To my understanding, first they say they did not find the difference from a slope of 1 and afterward state that this "(...) difference is further supported (...)". Which is it then?

We apologise for this confusion. Following on from the previous point, this again reflects the multiple comparisons of the fitted slopes: while the slopes of the starting point regressions are not significantly different from 1, they are higher than the slopes from the preceding regressions across all generations, shown by the lack of overlap in confidence intervals. We have amended the description of these comparisons to better convey this procedure.

Literature

Oskarsson, A. T., Van Boven, L., McClelland, G. H., & Hastie, R. (2009). What’s next? Judging sequences of binary events. Psychological Bulletin, 135(2), 262–285. https://doi.org/10.1037/a0014821

Williams, J. J., & Griffiths, T. L. (2013). Why are people bad at detecting randomness? A statistical argument. Journal of Experimental Psychology: Learning, Memory, and Cognition, 39(5), 1473–1490. https://doi.org/10.1037/a0032397

Nickerson, R. S. (2002). The production and perception of randomness. Psychological Review, 109(2), 330–357. https://doi.org/10.1037/0033-295X.109.2.330

Schulz, M.-A., Schmalbach, B., Brugger, P., & Witt, K. (2012). Analysing Humanly Generated Random Number Sequences: A Pattern-Based Approach. PLOS ONE, 7(7), e41531. https://doi.org/10.1371/journal.pone.0041531

Annand, C. T., & Holden, J. G. (2022). Embodied nonlinear dynamics of cognitive performance. Journal of Experimental Psychology: General. https://doi.org/10.1037/xge0001319

Biesaga, M., Talaga, S., & Nowak, A. (2021). The Effect of Context and Individual Differences in Human-Generated Randomness. Cognitive Science, 45(12), e13072. https://doi.org/10.1111/cogs.13072

Biesaga, M., & Nowak, A. (2024). The role of the working memory storage component in a random-like series generation. PLOS ONE, 19(1), e0296731. https://doi.org/10.1371/journal.pone.0296731

Vandierendonck, A., Demanet, J., Liefooghe, B., & Verbruggen, F. (2012). A chain-retrieval model for voluntary task switching. Cognitive Psychology, 65(2), 241–283. https://doi.org/10.1016/j.cogpsych.2012.04.003

<u>Reviewer #2</u>

This paper studies the kind of mental sampling process that has become increasingly influential in various models of decision making. The authors develop an experimental paradigm with choice between risky gambles in which the (typically latent) samples representing the possible outcomes are explicitly elicited. Across two online experiments (total N = 103), participants repeatedly generated potential outcomes from pairs of monetary gambles before choosing between them. The authors used these “random generation” sequences to infer how internal sampling might deviate from objective probabilities, serial independence, or have biased started points. In relation to their generated sequences, they find that participants over-generated rare outcomes, under-generated common ones and avoided repeating values. In relation to choice, they find no significant difference for nearly all decision algorithms in predicting actual participant choice when using the generated sequences than when using the described objective probabilities, except for the ‘higher mean’ algorithm, which was significantly greater when using the participant generated sequences.

The studies are novel and endeavor to bear on a general class of models, which speaks in their favor. The writing is clear, and the analyses are generally well performed. I do have some issues with the interpretation of results, which substantially limits their meaning and applicability, and should be recognized before the paper is ready for publication.

The fundamental assumption upon which the most interesting claims of this paper rest is that participant reported sequences actually mirror the mental sampling that might be taking place. However, it is not clear that this is the case here. Presumably, mental sampling particularly of simple distributions is much faster than participants are able to report in this experiment, and there is much more that goes into participants finally reporting the samples. Perhaps the reported samples are more about communication – participants are trying to

communicate the distribution to another person via the samples? Perhaps is it about trying to emulate randomization – participants are trying to generate samples that seem to be random for the specified distribution? Both of these processes are quite separate from "mental sampling". If there were a stronger correspondence between mental sampling and the reported sequences, I think we would see stronger results predicting actual choice. This is a major limitation of the study and the authors should discuss it in much more depth. Assuming that these reports aren't at all related to actual mental sampling in the decision process, how would we interpret the results of the study? What is its worth? Assuming it is noisy, but bears some resemblance to actual mental sampling, how might we interpret the results then?

This is an excellent question. As you say, our study does operate on the assumption that random generation tasks provide insight into mental sampling processes, though we do not mean to claim that explicit generations are one-to-one reports of actual mental samples; rather, we hope that these responses could illustrate the key signatures of the underlying sampling algorithm to help discriminate existing models of this process, and we now note this more directly in the Introduction. This does add a layer of obfuscation, though this is true of any attempt to trace this process: mental samples are ultimately unobservable, meaning these must be inferred, inevitably introducing some uncertainty. In this regard, part of the contribution of our paper is to examine this link through the association between generations and choice: while our previous submission showed some limited evidence of a connection in the match to particular choice rules, we now perform a more sophisticated regression analysis in the revised manuscript, finding generations do provide additional predictive ability. Moreover, while our task may not completely capture the full decision process, by revealing patterns in the sampling algorithm it can still reflect a key part of it: even if explicit generations are not direct reports, these responses may still be representative of the internal evidence guiding choice, displaying similar patterns.

It is indeed possible that there are other influences on these responses, and we have added some elaboration of these interesting alternate readings of behaviour to the Discussion, though we do remain cautious in accepting post-hoc explanations of behaviour based on observed data patterns. We have also added discussion of the value of our study if this link is absent: even if generations do not capture the samples used to guide choices, these can still reveal the broader accessibility of choice outcomes in the mind, and the features of the sampling process. In addition, explicit samples may still inadvertently colour perceptions of available options, as suggested by shift in preference between pre- and post-generation choices in Experiment 2.

Many sampling-based theories of the decision making process are meant to represent processes where memory plays a big role, most notably query theory. However the task here uses numerical gambles, and presents the gambles throughout, therefore limiting the scope for memory biases. This doesn't completely ruin the premise since some models like DFT and other DDMs can be based on stimuli that are always present, but it may help explain why few distortions are found and why the samples have low added predictive power.

This is a good point: in fact, one of the reasons we used decisions from description rather than experience is to limit memory biases in order to focus more on the features of the underlying sampling process itself, and we now state this when outlining our Methods. While memory does likely play a substantial role in many decisions, this is not the only source of bias: a number of mental sampling models propose outcomes are sampled from descriptions using varying algorithms with their own particular biases (DFT, BEAST, UWS, DDMs), meaning our method is well-placed to provide valuable insight into these cases. It is also common practice to keep gambles on-screen throughout choice in decisions from description, so our design is not unusual in this regard. As you suggest, biases may indeed

be stronger when memory constraints are added, but the fact we still find biases without such effects is arguably a strength of our study. We do however appreciate that results could differ if generating from experience; we have therefore added a new paragraph to the Discussion outlining this possibility, and offering contrasts with generation from experience as a valuable future direction.

Because of the setup, I suspect that a number of the observed findings are due to a different unmentioned mechanism that has to do with communicating the distribution. Take the case where one outcome has a 1% probability of occurring. Given that people generate about 25 samples, the closest option to reflect the true distribution would be to report 0 samples. This feels obviously lacking when one is trying to convey the distribution to others. A participant may then sensibly throw in a single sample to demonstrate that it exists, even though this will look like overestimation of low probability events. So it may not indicate an error in probability judgment but a property of communicating representative samples. This notion would be consistent with the tendency to start generating high probability events for example. It would drastically alter the interpretation of results, and think this must be mentioned centrally.

This is indeed a plausible explanation of the results, though we would add that our task gave no reason to encourage such a communication reading: our experimental instructions framed generations as imagined outcomes of each gamble as if it were repeatedly played out, aiming to capture how outcomes naturally come to mind. We made no reference to summarising the gamble for an unknown third party so there was no particular reason participants would have made this assumption, unless it is some default approach to generation (though this would itself be a strong claim). Of course, we cannot be absolutely sure that participants answered in the exact manner we intended, though we also do not wish to commit to any post-hoc interpretation of the observed behaviours without more firm evidence. We have however added a new paragraph to the Discussion outlining this explanation, and thank the reviewer for this suggestion.

A few papers/literatures are perhaps relevant but not cited. Ronayne and Brown (2017, Journal of Mathematical Psychology) explicitly elicit the distributions of items in multi-attribute choices based on the choice set. There is also a sizeable literature in experimental economics on generation of random sequences (pertaining to mixed strategies in game theory) reviewed in Camerer (2003). This could also help explain some of the underrepetition if people are trying to convey randomness.

Thank you for these suggestions: indeed, the Ronayne and Brown (2017) paper uses a similar approach to ours, and we now include this citation. The literature on random generation in competitive settings is also interesting, but is somewhat different from the aims of our study: we use random generation to try to access the inherently stochastic mental sampling process, whereas this previous literature focuses on randomness as a method to counter anticipation by an opponent. We have however added a brief mention of this work in the Introduction to illustrate previous uses of these types of tasks.

Other comments:

- Was the number of about 25 samples generated for 2 gambles or each one in a trial? More explicit details on the elicitation process would make things clearer, even though they are not complicated.

Good question: the listed values were per gamble, meaning participants produced around 50 samples per pair; we now clarify this in the text. As suggested, we have added more detail on the task instructions to our Methods.

- If the responses for singular outcomes should always be the same, did everybody left after exclusions indeed follow this?

Yes: our task prevented participants from submitting invalid outcomes, and our exclusion criteria ensure this is upheld. We have added a note on this to the text.

- In Figure 2 what do the conditional medians look like?

Apologies, but we are uncertain exactly what is meant by ‘conditional medians’ here - this could simply be the median generation rate within each true probability rather than the means in case of pull from extreme values, though please note that this would only be a visual change to the figure: our regressions consider all individual data rather than the aggregates, and the plotted slopes are taken from these models. The ‘conditional’ label could however instead refer to medians conditional on the predictions of the regression models, though a cursory search suggests these may also be related to quantile regressions. Given this uncertainty, we are unsure precisely what is requested here, but are happy to offer more detail if needed.

- It would help readers to explain the autocorrelation function calculations in more detail, maybe with an example.

We have added greater detail on the ACF calculations, including definitions of ACF coefficients and theoretical standards for independence. Providing a succinct example of this process is difficult given that our analyses use 1000 shuffled variants of a given series, but we have amended the description of our analyses here to make this more accessible.

- Figure A1 is interesting and should at the least be mentioned more prominently in the main text.

Thank you for this suggestion: we placed these analyses in the appendices as we did not wish to cause confusion with the non-significant effects of outcome value in our main regressions. We have however expanded our description of these analyses in the main text, with the caveat that our main regressions still ultimately provide a more robust test as they account for correlations between value and probability (note that these supplementary analyses are now in Appendix B in the revised manuscript).

- How well do the samples predict choice for the pre-sample choice? Maybe I missed it, but it isn’t clear to me whether, for experiment 2 it is the pre or post score that is reported in the paper. I’m assuming it’s the post to be the same as experiment 1 – but regardless it’d be good to see both.

This is an interesting question: as suggested, the analyses reported in our previous submission focused on post-generation choice as these have a clearer direction of influence, though if pre-generation choices were based on a similar sampling process then behaviour could be similar. We have added a comparison of the rule set on pre-generation choice to Appendix D, finding none of the considered rules offered reliable predictions in this block. This further leads to our speculation that explicit generation may alter choices by encouraging participants to consider a broader set of evidence: generations may illustrate the general properties of the sampling algorithm, but samples taken for more natural choices may be restricted by lower counts or shorter duration.

- The authors should consider discussing the methods before the results.

Thank you, as suggested we have moved the Methods to precede the Results, which we believe aids comprehension.

Reviewer #4

While many decision making models assume some form of mental sampling process, the specifics of this sampling process often remain debated. The article suggests accessing the internal sampling process through the explicit generation of outcomes. The authors present a series of analyses on how people generate outcomes from described risky gambles and how these generated outcomes relate to subsequent choices. Participants generated more low-probability outcomes and fewer high-probability outcomes than warranted by objective probabilities. Results on alternation align with previous work on random generation of binary sequences, showing that people's generated outcome sequences tend to deviate from truly random processes. Yet, the predictive power of generated outcomes on choice—instead of true outcome probabilities—seems to be minimal.

The article is well-written, the analyses seem appropriate and the idea to link mental sampling processes with random generation of outcomes—in particular, from described risky gambles—seems to be a novel contribution to the literature. That being said, there are some major concerns about the underlying hypotheses, theoretical assumptions, and study design that may limit the contribution of the article to the field.

1. The research question is rather vague and leaves some room for interpretation about the goal of the study ("The current paper thus uses random generation to access the sampling process underlying decision making", p. 5, line 89). Similarly, there are no clearly stated hypotheses. If this study is, indeed, fully exploratory in nature, it might make sense to state this clearly and possibly collect more data to substantiate the evidence given specific hypotheses.

Thank you, this is an important point, also raised by Reviewer 1 above: the research questions considered in our paper are based on the mechanisms assumed in previous mental sampling models, and our analyses sought to evaluate these assumptions without fully committing to any particular model. We now specify these research questions at the end of the Introduction to make the aims of our study more concrete.

2. The authors' main conclusion seems to be that the presented "findings suggest systematic biases in mental sampling" (p. 2, line 25-26). As such, the study builds on the assumption that random generation can appropriately trace mental sampling processes. However, the article is missing explanations and/or the discussion of (previous) evidence that convincingly establishes this relationship. Moreover, the authors state the possibility that "explicit generations [...] do not reflect the implicit evidence considered by decision makers [...] (p. 18, line 366-378) and continue to say that this is even implied by their results. Together, this seems to be a bit confusing: (1) can the authors convincingly establish the assumed relationship between mental sampling and random generation, or (2) did they a priori expect discrepancies between random generation and mental sampling, or (3) is this even the research question they set out to explore? The article may serve as an interesting contribution to clarifying the relationship between random generation and mental sampling, but this may require considerable reframing of the introduction.

These are excellent questions, also raised by Reviewer 2 above. It is indeed an assumption that random generation is able to trace mental sampling, though this is an unfortunate necessity when investigating unobservable mental processes: such mechanisms must be inferred from other measures, forcing a reliance on assumptions at some point. This also means that definitive evidence on this association is likely impossible, though this makes our

study even more valuable: by using explicit generations to predict choice, our paper offers an assessment of this link, finding a modest but reliable relationship. At the same time, we acknowledge that the uncertainty on this link remains a major limitation of our study, and the given quotes from our Discussion were intended to capture this. We now explicitly label this text as a ‘Limitations’ subsection in our Discussion.

Regarding our expectations for the study, we have amended our Introduction to clarify that we did not expect explicit generations to exactly match mental samples, but hoped that these could illustrate the features of the underlying sampling algorithm, allowing for discrimination of existing mental sampling models. This was certainly the original intention of our study: our tasks were specifically designed to provide series of generated outcomes that can be tested for particular signatures of the underlying sampling algorithm, and we now explicitly state the research questions targeted by our study at the end of the Introduction.

3. The authors highlight results from Experiment 2 as a novel contribution, suggesting that asking people to randomly generate outcomes alters their choices (from a pre- to a post-test). To substantiate this claim and justify any conclusions that follow from it, the analyses require an active/passive control condition.

This is a fair point: we did not wish to run experiments without random generation as this is the major methodological contribution of our study, and pure choice tasks are already dominant in the existing literature, but we appreciate this limits our interpretations of this difference. We have added an equivalent comparison using the data from Erev et al. (2017) which collected repeated choices from the same gamble pairs used in our experiments but with no intervening generations: this finds no similar increase in riskiness, further supporting our interpretations.

4. Information on the mechanistic aspects of how people generate outcomes from described probabilities is largely omitted in the article. However, this could be an interesting aspect that may be worthwhile to elaborate on and that could more explicitly inform the random generation–mental sampling relationship, as well as the references to different computational cognitive models. What cognitive processes do the authors suggest are at play?

This is indeed an interesting question, but potentially beyond the scope of our paper: the mechanism by which samples are generated from described distributions is assumed within existing mental sampling models rather than being proposed by our study, with different models assuming different processes (e.g., mental simulation, allocation of attention, memory retrieval). Our generation tasks do lean closer to a mental simulation reading, but are not intended to separate these mechanisms, focusing on the features of the sampling algorithm rather than its exact implementation. We have however added a paragraph elaborating on this point to the Discussion.

Minor

- The 2-minute time limit for random generation and restriction to generate outcomes in an alternating fashion make sense from a practical perspective, but could have considerable effects on the results. Given the work on optional stopping in sampling paradigms, it could be interesting to compare this condition with one that allows people to generate as many outcomes as they think best represent the gamble and to allow for non-alternating outcome generations.

This is a very good point: allowing participants to stop at any point could produce different patterns, but would likely restrict the number of samples collected, raising concerns that any findings are due to limited sample size rather than biased sampling itself. Of course, this

may be a more valid reflection of actual choice behaviour if most decisions are in fact based on small samples, but our goal here is to identify features of the underlying sampling algorithm given the differing assumptions of existing mental sampling models. We have added greater elaboration of this reasoning to our Methods, and noted optional stopping variants of our task as valuable future directions in the Discussion.

- Described risky gambles are arguably not extremely common in people's everyday lives. Without experience with what random outcome sequences of these risky gambles may look like, people could draw on their own (biased) experiences of random processes, which may hold some properties that are different from risky gambles (e.g., autocorrelation, sequential dependencies) and some that may be similar to the risky gambles used in this study (e.g., negative risk-reward relationship). Literature on the description–experience gap and more plausible statistical structures of real-world environments might be helpful starting points to put some of the presented findings in context beyond risky gambles with described probabilities.

This is a good point: we chose to use decisions from description to reduce the influence of memory on generations to focus on biases within the sampling process itself, but we appreciate that decisions from experience are also an important consideration. It is indeed possible that some of the biases we observe result from generalisation from outside experiences, though this links to the question of how samples are taken from descriptions raised above, which again our study wasn't intended to answer. We have added a new paragraph to the Discussion elaborating on further contrasts with generation from experience as a valuable future path for this work.

- We would like to encourage the authors to be a bit more specific in explaining the performed analyses, in particular, the random effects structure of the regression models (i.e., participant-specific slopes and intercepts) and how the (in-)dependent variables in these models came about (particularly if not explained in the CSV_guide.txt). For instance, it seems unintuitive that "start rate" is not an actual rate but a newly created binary variable (the label is particularly confusing in Figure 2), which does not seem to be explained in the manuscript. A dedicated section in the "Methods" part could maintain the brevity of the results section if needed.

We have added additional detail on the regression analyses to clarify that random intercepts and slopes were included for all predictors for each participant in all of our models. We have also added greater detail on our variables to the 'Design and Materials' section, and edited the Results to more concretely introduce each dependent variable. Regarding Figure 2, we used the label 'start rate' as this variable reflects the proportion of sequences in which an outcome was first generated, which for a single observation can only be 1 or 0; we have retained this label as we believe it better reflects the mean rates across participants, and amended the text describing this measure for clarity. We do not list such calculated variables in the CSV guide on our OSF page as this file reports only measures taken directly from participants (i.e., generated values, choices, and associated response times), though all our considered variables are defined in the analysis files themselves.

- The caption in Table 1 could benefit from some more explanation, for instance, that "B choice rate" refers to the risky option, whereas "A" is the safe option in most cases, except for Gamble 2 where both options are risky.

This is a good suggestion: we now state in the caption of this table (now Table 3) that the listed choice rates specifically reflect preference for riskier options.

- Table 2: Please describe the numbers under "A" and "B" as outcome and probability in the caption.

We have added a definition of the gamble format to the caption of this table (now Table 1).

- Please indicate the age unit (here: year). Given the large age range and previous work pointing to age differences in preferential choice, for instance, in decision quality and risk aversion, it might be informative to include age as a covariate. Otherwise, this may be an issue worth discussing.

As suggested, we now specify age is given in years. While age might indeed be an interesting covariate, demographic data for our samples was provided separately by the Prolific recruitment platform with no link to an individual's responses, so further analysis of any demographic factors on reported behaviour is not possible.

- Please spell out "CI" in the caption of Figure 4 to avoid confusion with credible intervals.

Thank you, we have amended this caption and others using this initialisation for specificity.

- Did you perform any attention checks and any measures to prevent bots/ people using bots from participating in your study?

No, our experiments did not include attention checks.

- Please add the unit of the reaction times to the data documentation. Looking at the reaction times, there seem to be some additional participants who might not have paid much attention to the task (many trials with generation reaction times of what seems to be well under 1 second, with streaks of alternating or the same outcomes).

Thank you, we now specify in our documentation that all response times are measured in seconds. We chose not to exclude data based on response time as we found during pilot testing that participants were able to answer surprisingly quickly, making it difficult to set a reasonable minimum. In addition, the 'Z' and 'M' key combination used to confirm responses provides some assurance that such patterns were deliberate by preventing participants from simply hammering the same number keys.

COMMSPSYCHOL-25-0744A Reviewer Responses

Editor

Dear Dr Spicer,

Your manuscript titled "Generated Outcomes in Risky Choice Reveal Biased Sampling and Sequential Dependencies" has now been seen by our reviewers, whose comments appear below.

Reviewer #4 has significant concerns regarding the evidentiary strength of the manuscript in its current form. We discussed these concerns with one of the other members of the panel of referees, who expressed the view that while the concerns were warranted, and that there are limitations the generalizability of the results, the insights that the work offers do make a significant contribution to the field. In revision, we require a more caveated presentation of the inferences supported by the results, but you are not required to add more empirical work.

In light of their combined advice we are happy, in principle, to publish a suitably revised version in Communications Psychology.

We therefore invite you to revise your paper one last time to address the remaining concerns of our reviewers, especially regarding the limitations of the approach used, and a list of editorial requests. At the same time we ask that you edit your manuscript to comply with our format requirements and to maximise the accessibility and therefore the impact of your work.

Thank you for providing us with a further opportunity to revise our paper. We are glad that you and the reviewers find value in our study, and are grateful to be able to answer the remaining concerns. As suggested, we have amended the text to better clarify our interpretations of our findings, add greater nuance to our conclusions, and further acknowledge the limitations of our approach. We hope that the paper is now suitable for publication with these changes, but are happy to make further edits if necessary.

EDITORIAL REQUESTS:

Please review our specific editorial comments and requests regarding your manuscript in the attached "Editorial Requests Table". Please outline your response to each request in the right hand column. Please upload the completed table with your manuscript files as a Related Manuscript file.

If you have any questions or concerns about any of our requests, please do not hesitate to contact me.

We have followed the requests from this form and hope the manuscript now follows the journal’s format requirements, but are happy to make further amendments as needed.

We hope to hear from you within two weeks; please let us know if you need more time.

Best regards,

Jennifer Bellingtier, PhD

Senior Editor

Communications Psychology

Caroline Charpentier, PhD

Editorial Board Member

Communications Psychology

orcid.org/0000-0002-7283-0738

REVIEWER EXPERTISE:

Reviewer #1: mental sampling, random generation processes

Reviewers #2 and #3 (co-review with ECR): mental sampling, risky decision-making

Reviewers #4 and #5 (co-review with ECR): risky decision-making

REVIEWERS' COMMENTS:

Reviewer #1

I am happy with the reviews done by the authors. All my concerns and doubts were addressed. Having said that, I would suggest that authors consider splitting the introduction into shorter sections. This is not a necessity since, as it is now, this reads well, but rather something to consider.

Thank you, we are glad our amendments to the paper were beneficial. As suggested, we have split the Introduction into shorter paragraphs to aid reading.

While I think the manuscript is suitable for publication, I would like the authors to address in the final version one detail that raised my concern when reading the reviewed manuscript. That is the description of the gratification for the participants. To my understanding, there was a fixed rate for the participation and a bonus within a range between £0 and £15 (the range is only explicitly stated in Study 1, but I assume in Study 2 it was the same). However, I am not sure how exactly the upper bound of the bonus was calculated. As far as I understand, it should be 5% of the highest possible win. According to Table 1, the highest possible win is £256, and consequently, the highest possible bonus is £12.8, not £15.

Thank you for this comment. You are correct that the true maximum possible bonus in these experiments was £12.80 - we listed the range as £0-£15 for simplicity of communication, but have now amended this for specificity. We have also added a specific note that the maximum realised bonus in both tasks was £2.50.

Moreover, the actual average of the bonus in Study 1 was £0.33 (so the sum of all bonuses was around £17), and in Study 2, it was £0.41 (so it adds up again to around £17). While I understand that money in science does not grow on trees, I would suggest in future studies to be more explicit about what the participants can expect because. In my opinion, it is a bit misleading to say that the bonus could be up to £15 while the actual chances of being even close to such an amount are negligible. Moreover, the total sum of the bonuses for all participants was only slightly bigger than the upper bound of the declared possible bonus…

This is a fair point: the distribution of potential bonuses in our tasks was highly skewed, with higher values being exceedingly rare, though this is a common feature of monetary gambles

given frequent negative correlations between risk and reward, including the gambles used here. In this regard, participants may have actually anticipated this given common awareness of this relationship in the environment (Pleskac and Hertwig, 2014), though we cannot say this with certainty. Statements of the anticipated reward in addition to the range may indeed be useful to set participants' expectations when deciding whether to take part in an experiment, though this should not impact their behaviour within the task itself as outcome probabilities within each gamble were precisely defined.

Reviewer #2

Thanks to the authors for a thoughtful revision. The difference between mental and reported samples has now been made more explicit in the paper. My comments are almost entirely addressed.

Thank you again for your feedback on the previous submission, we are glad our edits were satisfactory.

p. 31: "participants may have sought to use their generations to communicate the gambles' distribution of outcomes to a third party" It need not be a third party, if the experimenters themselves are the second party.

This is true, though if this were the case such communication is entirely unnecessary: the experimenter knows the gambles' distributions as they initially provided them. We have however removed the 'third party' term.

Regarding Figure 2, I did indeed mean the "median generation rate within each true probability rather than the means".

Thank you for the clarification: in this case, we refer back to our previous response that median rates do tend further towards the respective extremes of the scale, so more closely matching the true probabilities, but our statistical analyses use individual observations and so remain unaffected by this distinction.

Reviewer #4

We appreciate the effort invested in revising the manuscript and note clear improvements in several sections (e.g., the introduction on random generation, rule analyses, and the discussion). However, the revision remains largely superficial with respect to the central issues flagged previously. Two core concerns persist and make it difficult to establish strong claims: (a) conceptual clarity about the link between mental sampling and random generation, and (b) the adequacy and credibility of the evidence—especially the pre-/post-generation choice difference in Experiment 2.

Thank you for your feedback. While we are glad that our edits to paper were helpful, we are sorry that these did not fully resolve your conceptual concerns with our study. As detailed below, we have made further edits to the paper to better convey the relation between mental sampling and generation, and add greater elaboration on the limits on the evidence provided by our study.

The manuscript still treats random generation and mental sampling as more tightly linked than the evidence may warrant; the response letter and revisions do not yet resolve this

ambiguity. While it seems to be an interesting avenue to investigate whether explicit generation reflects mental sampling, parts of the manuscript read as if this link were already established (e.g., line 118: “The advantage of random generation in choice settings is that this procedure mirrors the mental sampling assumed in the decision making theories noted above”). Elsewhere, “random/explicit generation” appears to already be replaced by “mental sampling” (e.g., abstract, line 20: “These findings suggest systematic biases in mental sampling...” when referring to the results from the random generation task). Further conceptual inconsistencies arise from the findings of Experiment 2 and the discussion thereof. If random generation both mirrors mental sampling and causally alters subsequent choice, how should we understand the process underlying “pre-generation” choice? Does pre-generation choice entail no mental sampling, or is a qualitatively different sampling process involved, or does this pattern suggest that random generation is not an appropriate proxy for mental sampling more generally speaking? This would be important to clarify in light of the implications for the computational models the authors seek to evaluate.

We apologise for any continued conflation of explicit generations and mental sampling: as noted in our previous responses, we did not intend to suggest that our tasks definitively prove a link between these tasks, and we are sorry that the manuscript remained unclear on this. These are in some cases issues with open-ended language which can be interpreted in multiple ways: for example, our use of the word ‘mirror’ in the quoted sentence was not intended to suggest that generation and sampling are exactly matched, but simply that these processes resemble one another. We have amended the text in these cases to be clearer in distinguishing these tasks.

It is true that our findings could be attributed to differing sampling processes between implicit mental sampling and explicit generations, limiting the implications of our results for existing choice models, though as we note in our Discussion, there are also more basic differences in our tasks that may have also contributed to this difference (e.g., duration of the sampling period). We now highlight this issue more thoroughly in the Discussion, specifically noting the concerns raised by the pre- and post-generation differences, and clarifying that this may restrict our assessment of existing theories.

The re-analysis of secondary data (i.e., Erev et al., 2017) is a useful addition to the pre-/post-generation choice difference and may serve as a starting point to generate testable hypotheses. However, it is not sufficient to substantiate the claims implied by the observed pre-/post-generation choice difference, given substantial methodological and sample differences. The manuscript itself acknowledges that the present data may not yet yield the desired credibility (discussion, line 562: “further contrasts […] will be needed to confirm whether this is a reliable result”). We strongly encourage the authors to run separate control/experimental conditions that may provide more informative insights into the underlying process, for instance, whether the time between choices or extended exploration of the outcome space may provide more explanatory power for altering choice. When collecting additional data, it seems advisable to integrate standard measures for attention and bot checks.

This is a fair point: while our reanalysis of the existing data does provide an informative comparison point, we appreciate that more direct contrasts using more targeted designs are needed to fully verify any differences between pre- and post-generation choice. Based on the editor’s suggestions, we do not include such contrasts in the revised manuscript, retaining our focus on generation tasks, though we do now better acknowledge this issue when describing this difference in the Discussion, and will certainly consider this further in future continuations of this work.

Other comments

Exploratory status: The newly added research questions suggest there were no a priori formalized predictions. If so, please state clearly that the work is exploratory, so readers can appropriately weigh the evidence and the role of post hoc interpretation.

Thank you for raising this point. The distinction between exploratory and confirmatory research is somewhat blurred here: existing mental sampling models do provide expectations on sampling behaviours, but our paper avoids committing to these as specific hypotheses, instead identifying key factors across models which can be tested in our experiments. As such, our evaluations are not entirely post-hoc, so labelling the study as 'exploratory' would also be somewhat misleading.

Likelihood ratios vs Bayes factors: As noted (lines 484–485), the reported quantities are likelihood ratios, not Bayes factors (which require marginal likelihoods over priors). To avoid confusion, please label them "likelihood ratios" and, if desired, note their interpretive analogy to inclusion Bayes factors.

Thank you for catching this: indeed, as likelihoods were maximum estimates from the respective regression models for each rule, comparisons are likelihood ratios rather than Bayes factors. We have clarified this in the manuscript.

In sum, the manuscript has improved in presentation, but core conceptual and evidential issues remain unresolved. Substantial revision—particularly clarifying the construct mapping and providing new data—is necessary before strong conclusions can be drawn.

We appreciate these concerns with our study and hope our edits to the Discussion better acknowledge the limits of the provided evidence.
